# Comprehensive review of mapping climate change impacts on tea cultivation: bibliometric and content analysis of trends, influences, adaptation strategies, and future directions

**DOI:** 10.3389/fpls.2024.1542793

**Published:** 2025-01-24

**Authors:** Altyeb Ali Abaker Omer, Chun-Hua Zhang, Jie Liu, Zhi-guo Shan

**Affiliations:** ^1^ School of Tea and Coffee, Puer University, Puer, China; ^2^ Yunnan International Union Laboratory for Digital Protection and Germplasm Innovation Application of Tea Resource in China and Laos, Puer University, Puer, China

**Keywords:** tea cultivation, climate change, tea quality, antioxidants, secondary metabolites, adaptation strategies, sustainability, socioeconomic impacts

## Abstract

Climate change has a profound impact on *tea cultivation*, posing significant challenges to yield, quality, and sustainability due to stressors such as drought, temperature fluctuations, and elevated CO₂ levels. This study aims to address these challenges by identifying and synthesizing key themes, influential contributions, and effective adaptation strategies for mitigating the impacts of *climate change* on *tea production*. A systematic bibliometric and content analysis was conducted on 328 peer-reviewed documents (2004–2023), following the *PRISMA* methodology. Performance analysis using *Bibliometrix* examined trends in publication output, leading contributors, and geographical distribution, while science mapping with *VOSviewer* revealed collaboration networks and thematic clusters. A detailed review of highly cited studies highlighted the primary climate variables affecting tea cultivation and identified innovative adaptation strategies, as well as critical knowledge gaps. The results show significant progress in understanding the physiological, biochemical, and molecular responses of tea plants to climate-induced stressors, including antioxidant mechanisms, secondary metabolite regulation, and genomic adaptations. Despite these advancements, challenges remain, particularly regarding the combined effects of multiple stressors, long-term adaptation strategies, and the socioeconomic implications of *climate change*. The findings underscore the need for interdisciplinary approaches that integrate molecular, ecological, and socioeconomic research to address these issues. This study provides a solid foundation for guiding future research, fostering innovative adaptation strategies, and informing policy interventions to ensure sustainable tea production in a *changing climate*.

## 1 Introduction

Tea (*Camellia sinensis*) is one of the world’s most widely consumed beverages and a vital socioeconomic resource for millions worldwide, particularly in major *tea*-producing countries such as China, India, Sri Lanka, and Kenya. Beyond its cultural significance, tea cultivation is an essential component of global agriculture, contributing billions of dollars annually to the economy and supporting the livelihoods of millions of smallholder farmers. However, tea cultivation is particularly vulnerable to the impacts of *climate change*, as its growth, yield, and quality are closely linked to environmental factors such as temperature, precipitation, and soil health. Recent *climate variability*, including erratic rainfall patterns, prolonged droughts, and extreme temperatures, has intensified challenges in *tea* production, threatening the sustainability of this vital crop and the livelihoods of those dependent on it (Han et al., 2017; Wang et al., 2018).

Over the past two decades, research on the effects of *climate change* on tea cultivation has grown significantly, reflecting the urgent need to address these impacts. Prolonged drought and heat stress, for example, disrupt the synthesis of critical secondary metabolites such as catechins and flavonoids, which are crucial for *tea quality* (Guo et al., 2017). Similarly, extreme precipitation events degrade soil structure and nutrient availability, leading to long-term declines in productivity (Sahu et al., 2025). Despite advances in research, critical gaps remain. Many studies have focused on isolated variables, such as elevated CO₂ or single drought events, but the combined effects of multiple stressors—common in real-world scenarios—are underexplored (Jayasinghe and Kumar, 2019; Gu et al., 2023). Moreover, much of this work has been conducted in controlled laboratory settings, which fail to account for the complexities of field conditions, including soil health, altitude, and regional *climate variability* (Li et al., 2023). This disconnect limits the development of effective adaptation strategies for *tea cultivation*.

Emerging research highlights the potential of molecular and biochemical tools to enhance tea plants’ resilience to *climate stressors*. Advances in omics technologies, such as transcriptomics, metabolomics, and proteomics, have identified essential genes and pathways associated with stress adaptation. For instance, studies have shown that GRAS family transcription factors and microRNAs (miRNAs) regulate drought and heat tolerance, offering promising targets for genetic improvement (Zhao et al., 2022; Ashraf et al., 2023). Metabolomic studies have further revealed how stress-induced shifts in catechin and theanine pathways influence plant health and tea quality, providing valuable insights for sustainable cultivation practices (Han et al., 2017). However, these findings often lack field validation, and their application in real-world farming contexts remains limited.

In addition to molecular research, *climate modeling* offers valuable insights into the future of *tea cultivation*. Models predicting temperature and rainfall variability forecast significant shifts in the suitability of tea-growing regions, particularly in low-elevation areas, underscoring the need for adaptive measures (Thankappan, 2023; Jayasinghe and Kumar, 2019). Yet, these predictions are rarely translated into actionable policies or practices that smallholder farmers—who form the backbone of the tea industry—can implement. Economic and informational barriers further exacerbate farmers’ vulnerability, as adaptive practices such as water management, soil conservation, and the adoption of resilient cultivars are often underutilized (Wang et al., 2018).

Recent studies illustrate both the progress made and the persistent gaps in this field. For example (Ramírez-Gottfried et al., 2023), highlighted the benefits of compost tea for improving soil health and plant disease resistance, though its effectiveness under *climate stressors* remains unexplored (Li et al., 2023). emphasized the role of flavonoids in enhancing stress resilience but did not connect these findings to practical, field-level applications. Similarly (Liu et al., 2024), and (Fadhlina et al., 2023) explored the health benefits of tea, including anti-obesity and cholesterol-lowering properties, without addressing how climate variability affects the production of bioactive compounds (Chen et al., 2023). advanced molecular insights through omics technologies but did not integrate these findings into practical farming solutions (Elisha and Viljoen, 2021). identified sustainability gaps in rooibos tea cultivation but provided limited guidance for the broader tea industry. These studies highlight the fragmented nature of current research and the need for a comprehensive, interdisciplinary approach.

This study addresses these critical gaps through a bibliometric and content analysis of 328 publications (2004–2023). By synthesizing findings from diverse experimental and geographical contexts, this analysis explores the primary *climate variables* affecting *tea cultivation*, evaluates *adaptation strategies*, and identifies emerging trends and knowledge gaps. In particular, it bridges the gap between laboratory and field realities, integrates socioeconomic perspectives, and provides actionable insights for smallholder farmers. Furthermore, it connects *climate models* with practical agricultural policies, ensuring global relevance and applicability.

This study aims to address the following research questions:

1. What are the publication trends related to the impacts of *climate change* on *tea cultivation* from 2004 to 2023?
2. Who are the most influential authors, sources, and countries in this field?
3. What are the most influential documents on the impacts of *climate change* on *tea cultivation*?
4. What are the primary research areas within this domain?
5. What are the key themes within this domain?
6. What collaboration patterns and networks exist among researchers in this field?
7. What *climate variables* impact *tea cultivation* across different countries, and what *adaptation strategies* are being applied?
8. What emerging trends, knowledge gaps, and research directions can be identified to guide future studies?

This study advances the discourse on sustainable agriculture and *climate adaptation* by addressing these research gaps. It emphasizes the need for interdisciplinary approaches that integrate molecular, agronomic, and socioeconomic perspectives to develop effective and scalable solutions for *tea cultivation*. By providing a comprehensive analysis, this study offers a roadmap for future research and policy interventions, ensuring the sustainability of one of the world’s most important crops in the face of a changing climate.

## 2 Methodology

This methodology was designed to conduct a bibliometric analysis of *climate change* impacts on *tea cultivation*, systematically capturing, evaluating, and synthesizing research published over the past two decades. The approach aims to assess trends, identify key contributors, and map emerging research themes. Below is an overview of the data collection, screening process, bibliometric analysis, and final interpretation.

### 2.1 Data collection and search strategy

The data for this study was obtained from the *Scopus* database, selected for its broad coverage and high-quality peer-reviewed literature. The search was conducted on November 12, 2024, with the goal of identifying publications addressing the effects of *climate change* on tea plants (*Camellia sinensis*) and *tea cultivation* in general. A Boolean search strategy was employed to ensure comprehensive coverage, incorporating various terms related to *climate impacts* on *tea cultivation*.

The specific search query was formulated using the following terms in the TITLE-ABS-KEY fields: (“*tea plants*” OR “*Camellia sinensis*” OR “*tea cultivation*”) AND (“*climate change*” OR “*global warming*” OR “*temperature rise*” OR “*rainfall variability*” OR “*drought*” OR “*pest impact*” OR “*soil degradation*”).

Filters were applied to restrict the results to:

- Publication Year: 2004–2023.
- Document Type: Peer-reviewed journal articles (LIMIT-TO (DOCTYPE, “ar”)).
- Language: English (LIMIT-TO (LANGUAGE, “English”)).

This search returned 511 documents. The metadata of these documents, including author names, titles, abstracts, keywords, and citation details, was exported for further screening and analysis.

### 2.2 Screening and selection

**Figure 1:**
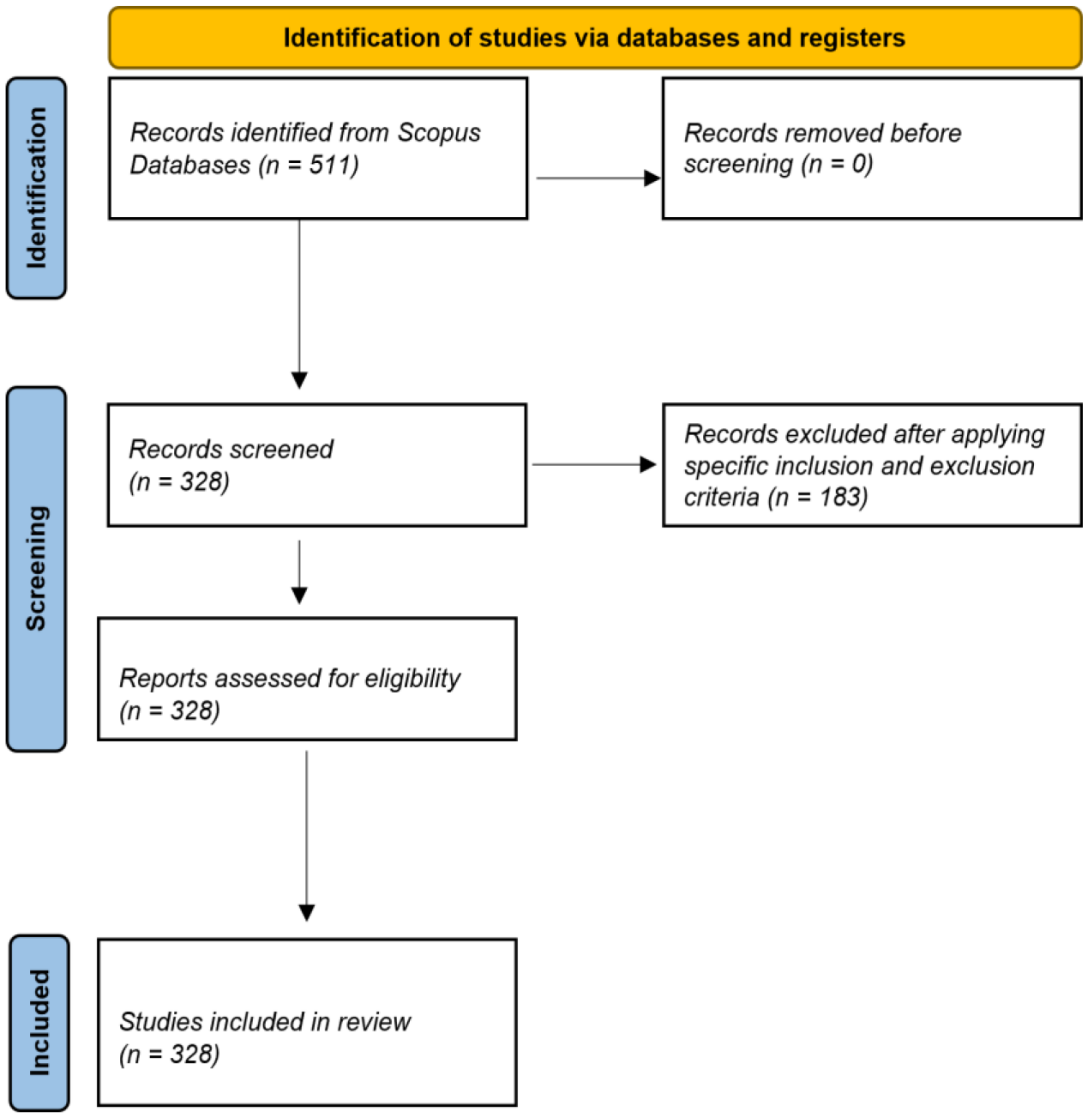
PRISMA framework flowchart.

To ensure the relevance and quality of the included studies, a rigorous screening process was applied following the *PRISMA* (Preferred Reporting Items for Systematic Reviews and Meta-Analyses) guidelines (Page et al., 2021). The initial filters were applied, followed by a second round of assessment, where the titles, abstracts, and keywords of the retrieved articles were reviewed to remove duplicates, irrelevant records, and confirm their alignment with the study’s objectives (**Figure 1**).

### 2.3 Inclusion criteria

The inclusion criteria were as follows:

1. The study must address the impacts of *climate change* on *tea cultivation*, focusing on environmental stressors such as temperature changes, drought, soil health, or pest dynamics.
2. Only *Peer-reviewed journal articles* focused on *tea cultivation* under *climate-induced stressors* were included.
3. Studies with only the *English* language were included.
4. Articles focused solely on the economic aspects, trade, or other unrelated crops were excluded.

In total, 328 documents met these criteria and were included in the final analysis.

### 2.4 Bibliometric analysis

- The bibliometric analysis was conducted in two stages: performance analysis and science mapping. Performance analysis: this was conducted using *bibliometrix* package version 4.3.0 in R (*biblioshiny*) (Aria and Cuccurullo, 2017) to extract descriptive metrics such as the yearly publication trends, influential authors, key journals, and geographical contributions. These metrics were compiled and visualized using *Microsoft Excel* to create tables and charts illustrating the evolution of research output over time. Notable findings included a significant increase in publications in recent years, reflecting heightened research activity in *climate change* and *tea cultivation*.
- Science mapping: science mapping was performed using *VOSviewer* version 1.6.20 software (van Eck and Waltman, 2010) to generate visualizations of collaboration networks and thematic clusters. Co-authorship analyses highlighted key contributors and their collaborative patterns, while citation analyses identified influential documents that have shaped the intellectual landscape. Keyword co-occurrence maps were created to reveal thematic clusters, illustrating research focus areas such as drought resilience, biochemical responses, and genomic adaptations. These visualizations helped demonstrate how the field has evolved and the relationships between central concepts and collaborative networks.

### 2.5 Visualization and interpretation

The results of the bibliometric analysis were visualized to provide a comprehensive understanding of the research landscape. The key outputs include:

- Annual publication trends and geographical insights: Charts illustrating publications growth over time and identifying major contributing countries, including *China, India*, and *Kenya*.
- Influential contributions: Tables and visualizations of the most cited documents, impactful authors, and leading journals in the field.
- Thematic and network analyses: Co-authorship, citation, and keyword co-occurrence maps that reveal thematic trends, such as increasing attention to omics technologies, stress physiology, and *climate adaptation strategies*.
- Content analysis: A detailed review of 25 highly cited studies to identify the primary *climate variables* impacting *tea cultivation*, *adaptation strategies*, and the emerging research directions guiding future studies.

By synthesizing trends, influential contributions, and thematic evolutions, the bibliometric approach used in this study provides a robust framework for understanding and addressing the impacts of *climate change* on *tea cultivation*. The visualizations and insights derived from this analysis will help identify knowledge gaps and guide future interdisciplinary research.

## 3 Results

### 3.1 Trends in research on climate change impacts on tea cultivation

**Figure 2:**
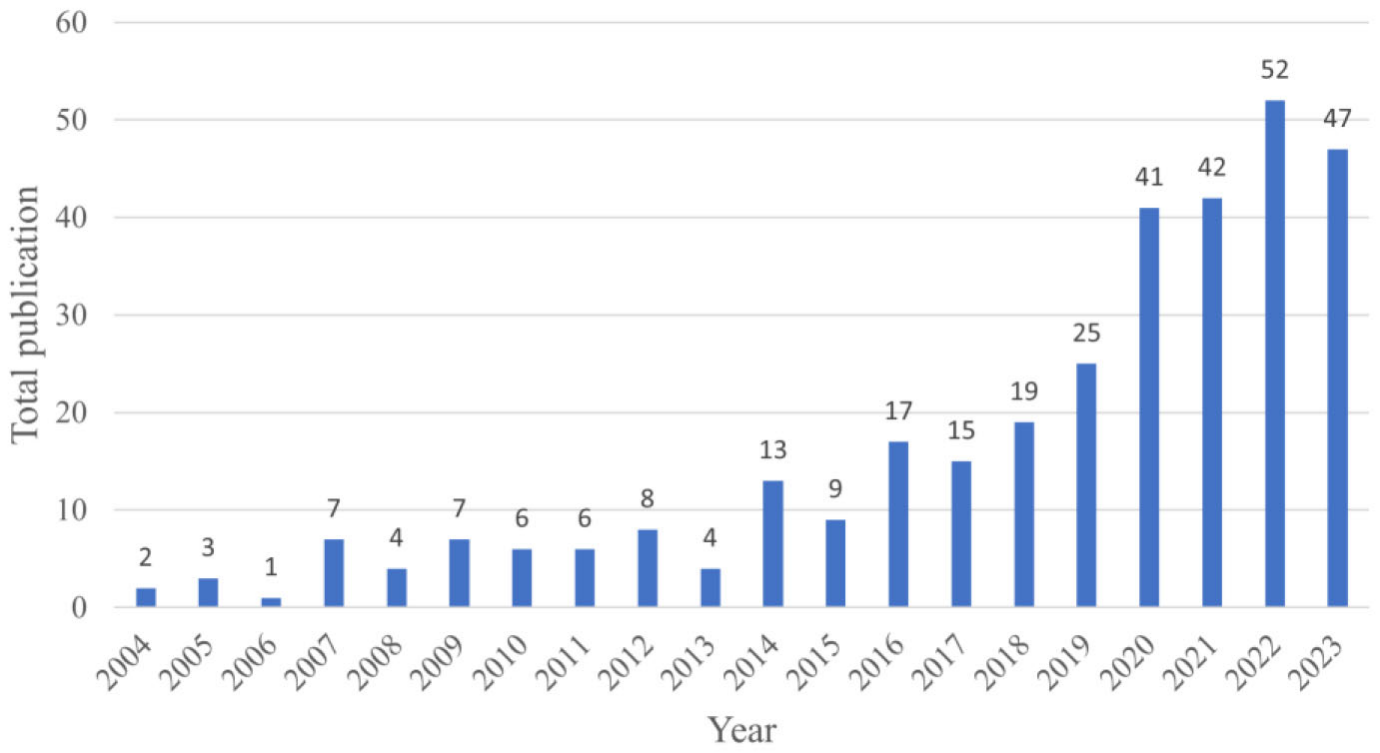
Annual publication trend (2004–2023) showing the growth of research on climate change impacts on tea cultivation, highlighting the steady increase in academic focus on this topic over time.

The chart (**Figure 2**) illustrates a clear progression in research on the impacts of climate change on tea cultivation, highlighting three distinct phases over the last two decades. From 2004 to 2013, research activity was minimal, with fewer than ten publications per year. This limited attention suggests that, during this period, the specific effects of climate change on tea were not yet recognized as a significant research priority. However, from 2014 onwards, there was a steady increase in publication numbers, reaching 19 by 2019. This rise likely reflects a growing awareness of the unique vulnerabilities of tea crops to climate variability, as well as a broader agricultural focus on understanding climate impacts. The most notable growth occurred from 2020 onward, with publications surpassing to over 40 annually and peaking at 52 in 2022. This surge in research intensity likely corresponds to the increasing climate challenges faced by tea-growing regions and a global push for sustainable agriculture practices. The consistently high numbers from 2021 to 2023 indicate that the impact of climate change on tea has become a well-established research focus. This recent body of work likely concentrates on adaptation strategies, crop resilience, and sustainable cultivation practices to support tea producers facing climate pressures. In summary, this upward trend reflects an evolving research field, responding to the urgency of climate impacts on tea cultivation and the growing need for actionable strategies to mitigate these effects.

### 3.2 Most influential authors, sources, and countries in climate change research on tea cultivation

#### 3.2.1 Most influential authors contributing to this field

**Table 1** lists the most influential authors in the field of climate change research on tea cultivation, ranked by their h-index, g-index, m-index, total citations (TC), number of publications (NP), and the year of their first publication (PY_start). These metrics provide a comprehensive view of each author’s impact, productivity, and career progression within this field.

**Table 1:** Top authors in climate change research on tea cultivation.

Author	h_index	g_index	m_index	TC^1^	NP^2^	PY^3^_start
Kumar Sanjay	15	15	0.75	737	15	2005
Ding Zhaotang	13	21	1.3	700	21	2015
Wang Yu	13	22	1.3	653	22	2015
Li Xinghui	11	12	1	793	12	2014
Wang Xinchao	11	13	1	402	13	2014
Yang Yajun	11	12	1	401	12	2014
Ahuja Paramvir Singh	9	9	0.529	546	9	2008
Han Wenyan	9	12	1	428	12	2016
Hao Xinyuan	9	10	0.818	356	10	2014
Singh Kashmir	9	9	0.529	546	9	2008

^1^ Total citations, ^2^ Number of publications, and ^3^ Publications by year.

*Kumar Sanjay* ranks highest in h-index and g-index, with an h-index of 15 and total citations of 737, reflecting a significant contribution since 2005. *Ding Zhaotang* and *Wang Yu* have equally strong profiles, with higher g-index and m-index values (1.3 each), highlighting their substantial influence through frequent and recent publications since 2015. *Li Xinghui* leads in total citations (793), indicating high impact, while *Wang Xinchao* and *Yang Yajun* maintain consistent productivity with high citation counts, both beginning their contributions in 2014.

Other influential authors include *Ahuja Paramvir Singh* and *Singh Kashmir*, both contributing foundational studies since 2008 with a steady citation rate. *Han Wenyan* and *Hao Xinyuan* have shown growing influence with substantial citation counts and high productivity since 2014 and 2016, respectively.

These authors represent the driving force in advancing knowledge on the impacts of *climate change* on tea cultivation. Their research contributes to various subfields, such as plant physiology, molecular adaptation, and environmental resilience. This analysis provides insight into the key contributors who have shaped the direction of research in this area and highlights potential collaborators for future studies.

#### 3.2.2 Most influential sources contributing to climate change and tea cultivation research

This section examines the most influential academic sources that have contributed significantly to the field of climate change impacts on tea cultivation, as identified by metrics such as the h-index, g-index, m-index, TC, NP, and the PY_start. The data in **Table 2** highlights journals that serve as primary platforms for disseminating research in this area.

**Table 2:** Leading academic sources in climate change research on tea cultivation.

Source	h_index	g_index	m_index	TC^1^	NP^2^	PY^3^_start
Frontiers in Plant Science	11	19	1.222	561	19	2016
Scientific Reports	9	10	1.125	497	10	2017
International Journal of Molecular Sciences	8	10	1.143	211	10	2018
Plant Physiology and Biochemistry	8	9	0.727	216	9	2014
Scientia Horticulturae	8	11	0.667	233	11	2013
Functional and Integrative Genomics	6	6	0.375	408	6	2009
BMC Genomics	5	7	0.714	193	7	2018
Peerj	5	5	1	115	5	2020
Science of the Total Environment	5	5	0.833	222	5	2019
Biologia Plantarum	4	4	0.19	164	4	2004

^1^Total citations, ^2^Number of publications, and ^3^Publications by year.

**Table 2** provides an overview of the leading academic sources, ranked by their respective bibliometric indicators, reflecting their influence and prominence in climate change and tea cultivation research. Journals like *Frontiers in Plant Science* and *Scientific Reports* have the highest h-index scores, indicating a substantial impact based on citations and publication volume. Notably, *Frontiers in Plant Science* has an h-index of 11, a g-index of 19, and an m-index of 1.222, signifying both productivity and consistent influence since its entry in 2016. Similarly, *Scientific Reports* demonstrates robust metrics with an h-index of 9 and a total citation count of 497, underscoring its role as a key outlet for innovative studies in this domain since 2017.

Other prominent sources include the *International Journal of Molecular Sciences*, *Plant Physiology and Biochemistry*, and *Scientia Horticulturae*, each showing high g-index and total citation values, reflecting their contributions to molecular and physiological studies in tea plants under climate stress. Older publications such as *Functional* and *Integrative Genomics* and *Biologia Plantarum*, which started contributing in 2009 and 2004, offer foundational insights into genomics and plant biology, supporting long-term research continuity.

This table highlights the diverse range of scientific journals that provide crucial knowledge, spanning plant science, environmental studies, genomics, and biochemistry. Their cumulative contributions help shape an interdisciplinary understanding of how *climate change* affects *tea cultivation* from molecular to ecological perspectives.

#### 3.2.3 Most influential countries in climate change research on tea cultivation

**Figure 3:**
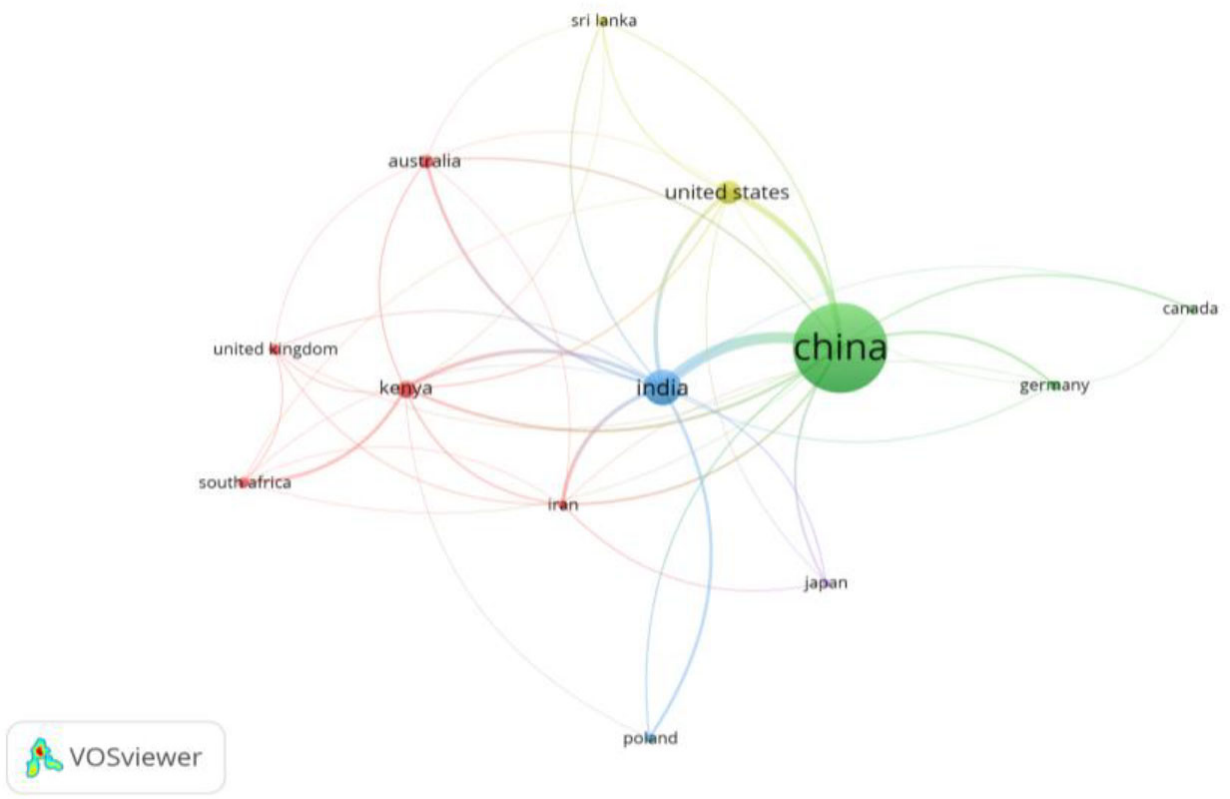
Network of influential countries in climate change research on tea cultivation, visualized through co-authorship and citation linkages. The size of each node represents the country’s research output, with larger nodes indicating higher publication and citation counts. Thicker lines denote stronger collaborative links between countries. Created with VOSviewer.

**Table 3** and **Figure 3** illustrate the most influential countries in *climate chang*e research on *tea cultivation*, ranked by TC, total publications (TP), and total link strength. *China* leads the primary contributor, with the highest number of publications (210) and citations (5160), reflecting its extensive research efforts in this domain. India ranks second, with substantial citation and publication numbers, underscoring its significant role in advancing research on climate-related impacts on tea.

**Table 3:** Top countries contributing to climate change research on tea cultivation.

Rank	Country	TC^1^	TP^2^	Total link strength
1	China	5160	210	150
2	India	1589	49	156
3	United States	972	26	57
4	Kenya	452	16	51
5	Poland	390	6	15
6	Australia	285	11	21
7	Sri Lanka	260	6	12
8	Canada	244	5	6
9	Japan	158	5	15
10	Spain	126	2	0
11	United Kingdom	125	8	10
12	Germany	124	7	13
13	South Africa	120	8	17
14	Iran	104	6	33
15	Serbia	63	1	0

^1^Total citations and ^2^Total publications.

The *United States*, *Kenya*, and *Poland* follow, demonstrating notable contributions in citations and collaborative strength. **Figure 3** provides a network view of these countries’ connections, highlighting prominent collaborations. *China* and *India* have strong linkages with other countries, facilitating knowledge exchange and collaborative studies on tea and *climate resilience*.

*Australia, Sri Lanka*, and *Canada* are also key contributors, while *Japan*, the *United Kingdom, Germany*, and *South Africa* play supportive roles in the global research network. Although their publication counts are lower, these countries contribute valuable insights, often through partnerships with leading research nations.

**Table 3** and **Figure 3** offer a comprehensive view of the countries shaping *climate change* research on *tea cultivation*, identifying the main contributors and their collaborative networks. This analysis highlights the global nature of tea research and underscores the importance of international cooperation in addressing *climate-related* challenges in tea-growing regions.

### 3.3 Geographical distribution of research on climate change impacts on tea cultivation

**Figure 4:**
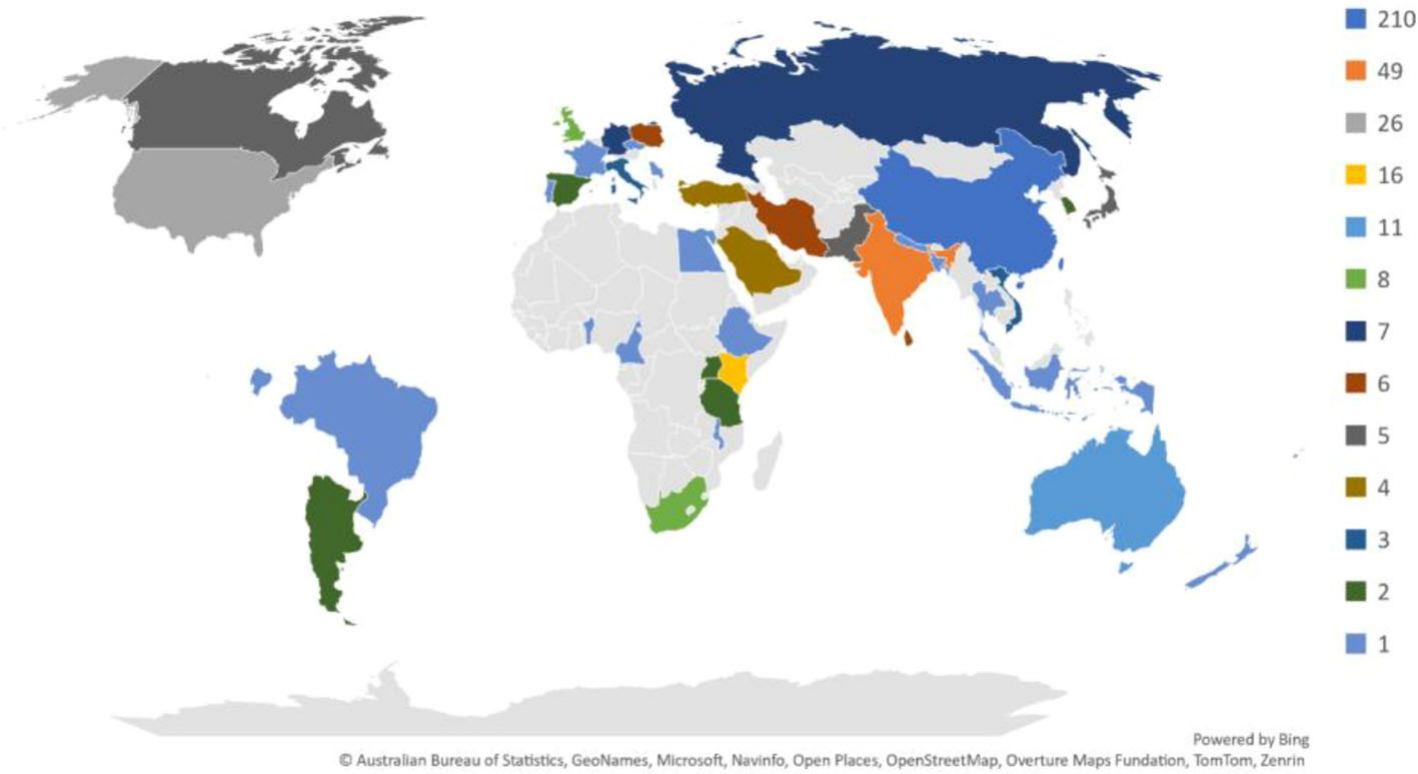
Global distribution of research publications on climate change impacts tea cultivation, represented by the number of studies per country. Darker shades indicate higher publication counts, reflecting the concentration of research efforts in specific regions.

**Figure 4** presents a geographical overview of the research output on *climate change* impacts on *tea cultivation* across various countries. The color gradient reflects the volume of research publications, with darker colors indicating a higher concentration of studies. This distribution reveals significant regional variation, with certain countries emerging as primary contributors to the field.

*China*, shown in dark blue, leads with the highest number of publications (210), underscoring its substantial investment in tea research and *climate adaptation strategies*. *India*, another major tea producer, has also produced a considerable volume of research (49 publications), highlighting its focus on understanding and mitigating the impacts of *climate change* on tea agriculture. Other countries with notable contributions include *Japan, Brazil*, and *Sri Lanka*, each producing substantial studies on the ecological, economic, and social impacts of *climate change* on *tea cultivation*.

In contrast, many tea-producing countries in Africa and Southeast Asia show limited research output on this topic, as indicated by lighter shades or the absence of color. This disparity may reflect differences in research funding, institutional focus, or available resources for studying climate impacts on agriculture in these regions.

This map provides valuable insights into the global research landscape on *climate change* and *tea cultivation*, identifying leading contributors and highlighting regions where further research could support *climate resilience* in *tea production*. This geographical analysis can help guide future collaborations and encourage knowledge-sharing initiatives to address global climate-related challenges in tea-growing regions.

### 3.4 Most influential documents on climate change impacts on tea cultivation

**Table 4** presents an analysis of the most influential studies on the effects of *climate change* on *tea cultivation*, ranked by total citations, citations per year, and normalized citation counts. These documents reflect critical advancements in understanding tea plants’ responses to climate-related stressors such as drought, temperature fluctuations, and CO₂ levels. Key studies include research on the biochemical pathways involved in stress tolerance, physiological impacts on tea quality, and genetic responses to environmental challenges.

**Table 4:** Key documents on climate change and tea cultivation, ranked by citation metrics.

Rank	Study	TC^1^	TC per Year	Normalized TC
1	(Singh et al., 2009)	192	12.00	3.44
2	(Li et al., 2019)	165	27.50	3.89
3	(Wang et al., 2016)	150	16.67	3.29
4	(Ahmed et al., 2014)	127	11.55	2.98
5	(Ahmed et al., 2019)	116	19.33	2.74
6	(Gai et al., 2020)	107	21.40	4.07
7	(Jayasinghe and Kumar, 2019)	104	17.33	2.45
8	(Zhou et al., 2014)	99	9.00	2.32
9	(Cheruiyot et al., 2007)	97	5.39	2.42
10	(Hernández et al., 2006)	95	5.00	1.00
11	(Wang et al., 2019)	89	14.83	2.10
12	(Upadhyaya et al., 2008)	87	5.12	1.47
13	(Liu et al., 2016)	81	9.00	1.78
14	(Li et al., 2017)	79	9.88	2.16
15	(Guo et al., 2017)	77	9.63	2.10
16	(Wang et al., 2018)	76	9.50	2.08
17	(Zhang et al., 2017)	76	9.50	2.08
18	(Han et al., 2017)	76	10.86	2.41
19	(Zheng et al., 2016)	76	8.44	1.67
20	(Singh et al., 2008)	75	4.41	1.27
21	(Sun et al., 2020)	74	14.80	2.81
22	(Wijeratne et al., 2007)	74	4.11	1.85
23	(Gu et al., 2020)	73	14.60	2.78
24	(Zou et al., 2014)	73	6.64	1.71
25	(Upadhyaya et al., 2011)	70	5.00	2.02

^1^Total Citations.

Prominent themes in these influential studies address a range of climate factors—drought, temperature extremes, and elevated CO₂—each impacting the biochemical composition and quality of tea. Some studies, for example, investigate how drought influences antioxidant compounds like *catechins* and *flavonoids*, which are crucial for tea’s flavor and health benefits. Others focus on the molecular and transcriptomic responses that enable tea plants to cope with environmental stresses, identifying potential genetic markers for resilience.

Through their cumulative citation impact, these documents collectively provide a foundation for understanding climate adaptation strategies in tea cultivation. They highlight research gaps, including the need for long-term studies on climate resilience and the application of genetic modifications for enhanced stress tolerance. This table underscores the importance of sustained research efforts to ensure the quality and productivity of tea under changing climate conditions.

### 3.5 Disciplinary distribution of research on climate change impacts on tea cultivation

**Figure 5:**
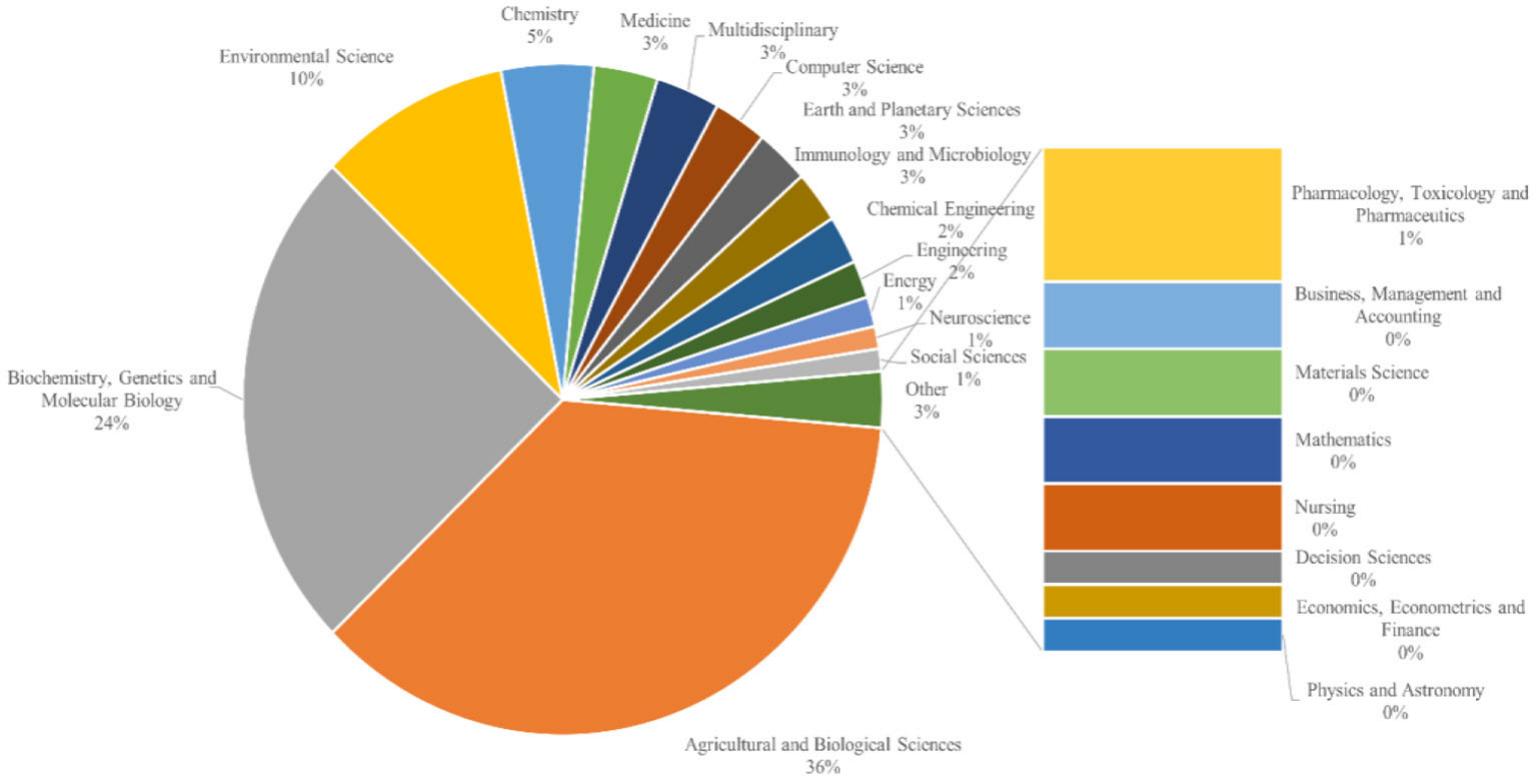
Disciplinary breakdown of research on climate change impacts on tea cultivation.

The pie chart (**Figure 5**) illustrates the distribution of research disciplines within climate change impacts on tea cultivation. The majority of studies fall under *Agricultural and Biological Sciences*, accounting for 36% of the research. This indicates a strong focus on plant science and agricultural practices. This is followed by *Biochemistry, Genetics*, and *Molecular Biology* at 24%, highlighting significant research on tea plants’ biochemical and genetic responses to climate change.

*Environmental Science* makes up 10% of the research, showing interest in ecological and environmental factors affecting tea cultivation. Smaller fields, such as *Chemistry* (5%), *Medicine* (3%), *Multidisciplinary Studies* (3%), and *Computer Science* (3%), represent niche areas that contribute to specific aspects of climate impact research on tea, such as chemical analysis, health-related studies, and data modeling.

Other fields, including *Immunology and Microbiology* (3%), *Earth and Planetary Sciences* (2%), and *Engineering* disciplines like *Chemical* (2%) and *Energy* (2%), highlight interdisciplinary contributions. Additional smaller contributions from *Neuroscience*, *Social Sciences*, *Pharmacology*, and *Business* also suggest that climate impact research on tea intersects with diverse disciplines, though with minimal representation in the field.

### 3.6 Primary research themes on climate change impacts on tea cultivation

**Figure 6:**
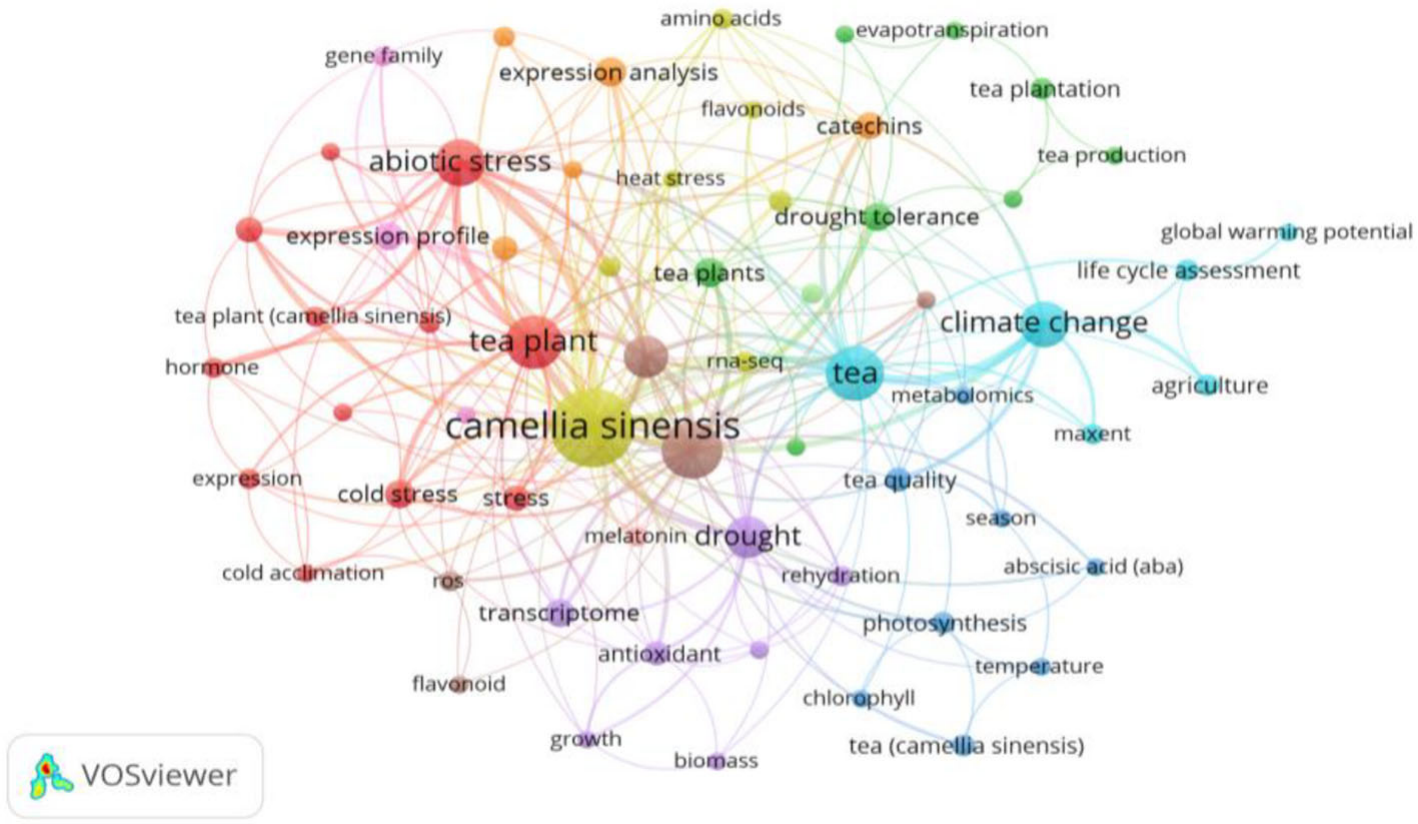
Author keyword co-occurrence network on climate change impacts in tea cultivation: This VOSviewer visualization showcases the primary research themes in climate change studies focused on tea cultivation, derived from author keyword co-occurrence analysis. Each node represents a keyword, with node size indicating the frequency of its appearance across studies, while the connections between nodes represent co-occurrence relationships. The colors represent distinct thematic clusters, each encapsulating a key area of focus within the research domain. Created with VOSviewer.

**Figure 7:**
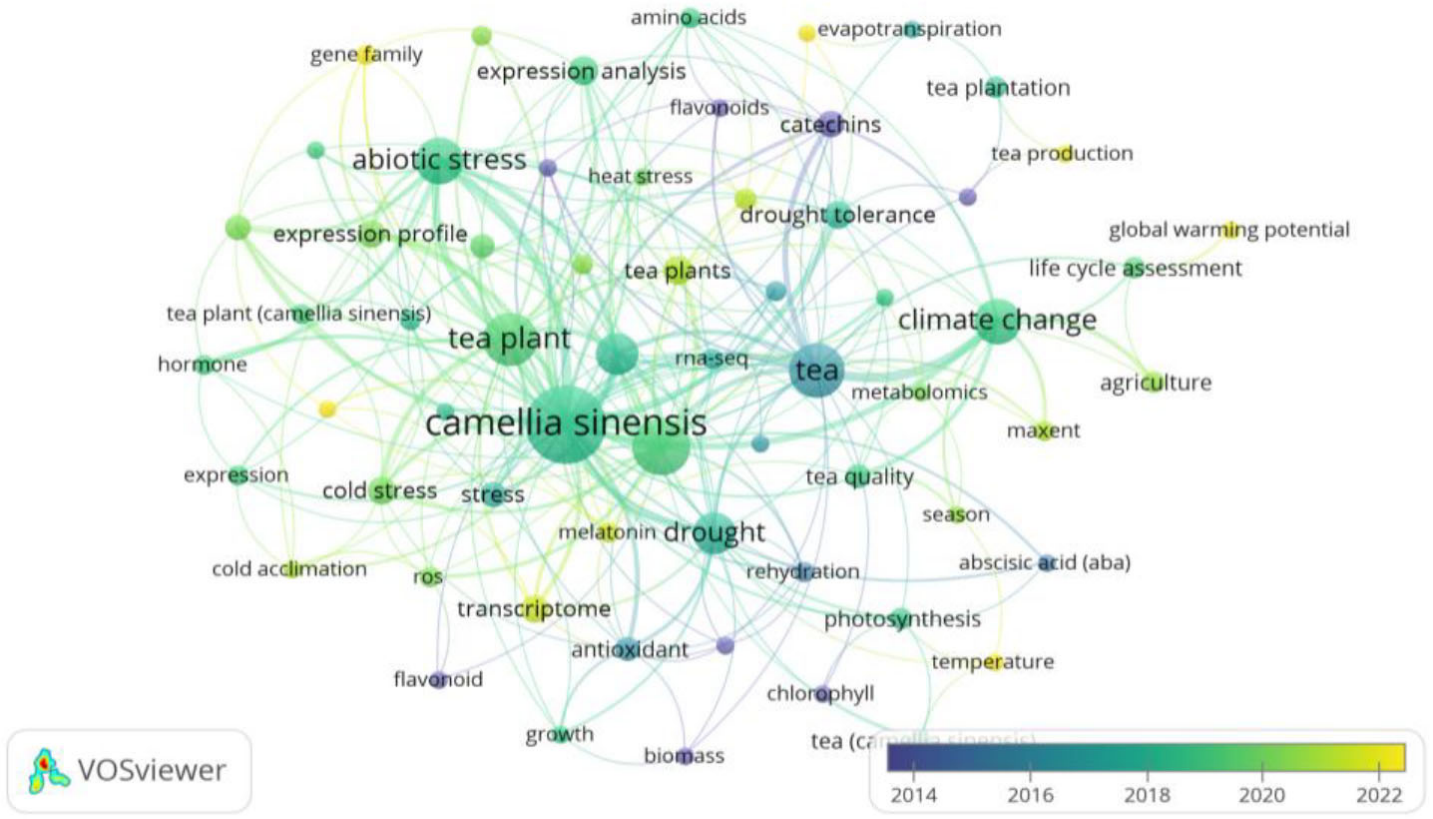
Temporal evolution of author keyword co-occurrence in climate change and tea cultivation research: This VOSviewer visualization presents the temporal development of research themes in climate change studies related to tea cultivation based on author keyword co-occurrence data from 2014 to 2022. Each node represents a keyword, with node size reflecting its frequency, while the color gradient from purple to yellow indicates the progression over time, as shown in the legend. Lines between nodes depict co-occurrence relationships, with thicker lines suggesting stronger associations. ‘Cold stress’ is prominently featured in the Abiotic Stress and Plant Response cluster (red cluster), linked with keywords such as ‘abiotic stress’, ‘expression profile’, and ‘transcriptome’. These associations underscore the focus on tea plants’ adaptations to low-temperature stress within the broader context of climate change resilience. Created with VOSviewer.

**Figures 6** and **7**, generated through *VOSviewer* visualizations, illustrate the primary research themes within the climate change impacts on tea cultivation derived from keyword co-occurrence analysis. These visualizations provide insights into interconnected research areas and the central focus of existing studies.

**Figure 6** presents an overview of keyword clusters, with each color-coded cluster representing related terms. The theme of *Abiotic Stress and Plant Response* appears prominently, with keywords such as “*abiotic stress*,” “*cold stress*,” “*expression profile*,” and “*hormone*.” This theme focuses on the physiological and hormonal adaptations of tea plants to environmental stressors, such as cold and drought, to enhance resilience in tea crops.

**Figure 7** adds a temporal dimension, showing the evolution of research themes from 2014 to 2022, as indicated by a color gradient. This figure highlights how themes like *Drought Tolerance* and *Water Management* have increased attention recently. Keywords within this cluster, including “*drought tolerance*,” “*evapotranspiration*,” and “*water stress*,” underscore the growing emphasis on improving water management practices for tea cultivation, particularly in response to climate-driven drought conditions.

Other prominent themes include Photosynthesis and Metabolomics, with keywords like “*photosynthesis*,” “*chlorophyll*,” “*temperature*,” and “*tea quality*.” This cluster addresses the physiological impacts of climate change on tea plant health and metabolic processes, focusing on how environmental changes affect tea quality and flavor.

Additionally, broader themes such as *Climate Change Impact* and *Adaptation Strategies* (e.g., “*climate change*,” “*global warming potential*,” and “*life cycle assessment*.”) explore the overall impacts of climate change on tea cultivation and seek sustainable adaptation practices to enhance the industry’s resilience. Finally, keywords related to *Biochemical Compounds* and *Antioxidant Properties* (e.g., “*antioxidant*,” “*catechins*,” “*flavonoids*”) and Genomic and Transcriptomic Studies (e.g., “*transcriptome*,” “*gene expression*”) indicate areas of research focused on understanding tea’s health benefits and genetic responses to stress.

Together, **Figures 6** and **7** provide a comprehensive view of the major research themes and trends in climate change studies on tea cultivation, highlighting well-established and emerging focus areas.

Prominent themes include the abiotic stress and plant response cluster (red cluster) prominently features the keyword *‘cold stress*,’ reflecting its critical role in the study of tea plants’ physiological and molecular responses to low-temperature environments. This keyword is closely associated with terms such as ‘*abiotic stress*,’ ‘*expression profile*,’ and ‘*transcriptome*,’ underscoring the importance of cold stress in understanding tea plants’ resilience to climate-induced stresses. Research within this cluster highlights various mechanisms, such as cold acclimation and antioxidant responses, which are pivotal for enhancing tea plant adaptability to adverse environmental conditions. The *Climate Change* and *Adaptation* theme (blue cluster) includes terms like “*climate change*,” “*life cycle assessment*,” and “*agriculture*,” reflecting the broader impact of climate variations on tea cultivation and sustainable practices.

Other significant clusters include *Photosynthesis* and *Metabolomics* (purple cluster), which examines metabolic responses and quality traits affected by climate factors, and *Water and Drought Tolerance* (green cluster), focused on terms such as “*drought tolerance*,” “*evapotranspiration*,” and “*water stress*,” highlighting water management and resilience strategies.

This network provides a comprehensive overview of interconnected research topics in climate change and tea cultivation, identifying both well-established and emerging themes. It underscores the field’s interdisciplinary nature, where molecular biology, environmental science, and agricultural practices converge to address the challenges climate variability poses on tea production.

Key research areas include *Abiotic Stress* and *Plant Response* (shown in green) with prominent terms such as “*abiotic stress*,” “*expression profile*,” and “*cold stress*,” highlighting a long-standing interest in the physiological adaptations of tea plants to environmental stresses. The *Climate Change Impact* and *Adaptation Strategies* theme (indicated by the blue cluster), with terms like “*climate change*,” “*life cycle assessment*,” and “*agriculture*,” demonstrates an ongoing focus on understanding and mitigating climate change effects on tea cultivation.

The color gradient reveals emerging themes, such as *Water and Drought Tolerance* and *Photosynthesis* and *Metabolomics*, gaining attention in recent years, with terms like “*drought tolerance*,” “*tea quality*,” “*photosynthesis*,” and “*temperature*” moving toward the yellow spectrum. This temporal view underscores the field’s evolving priorities, showing a shift toward resilience and sustainability in response to climate impacts.

This network provides a dynamic overview of the field’s research landscape, illustrating the established areas of inquiry and newly developing interests in studying climate change impacts on tea cultivation.

### 3.7 Collaborative networks in climate change research on tea cultivation

**Figure 8:**
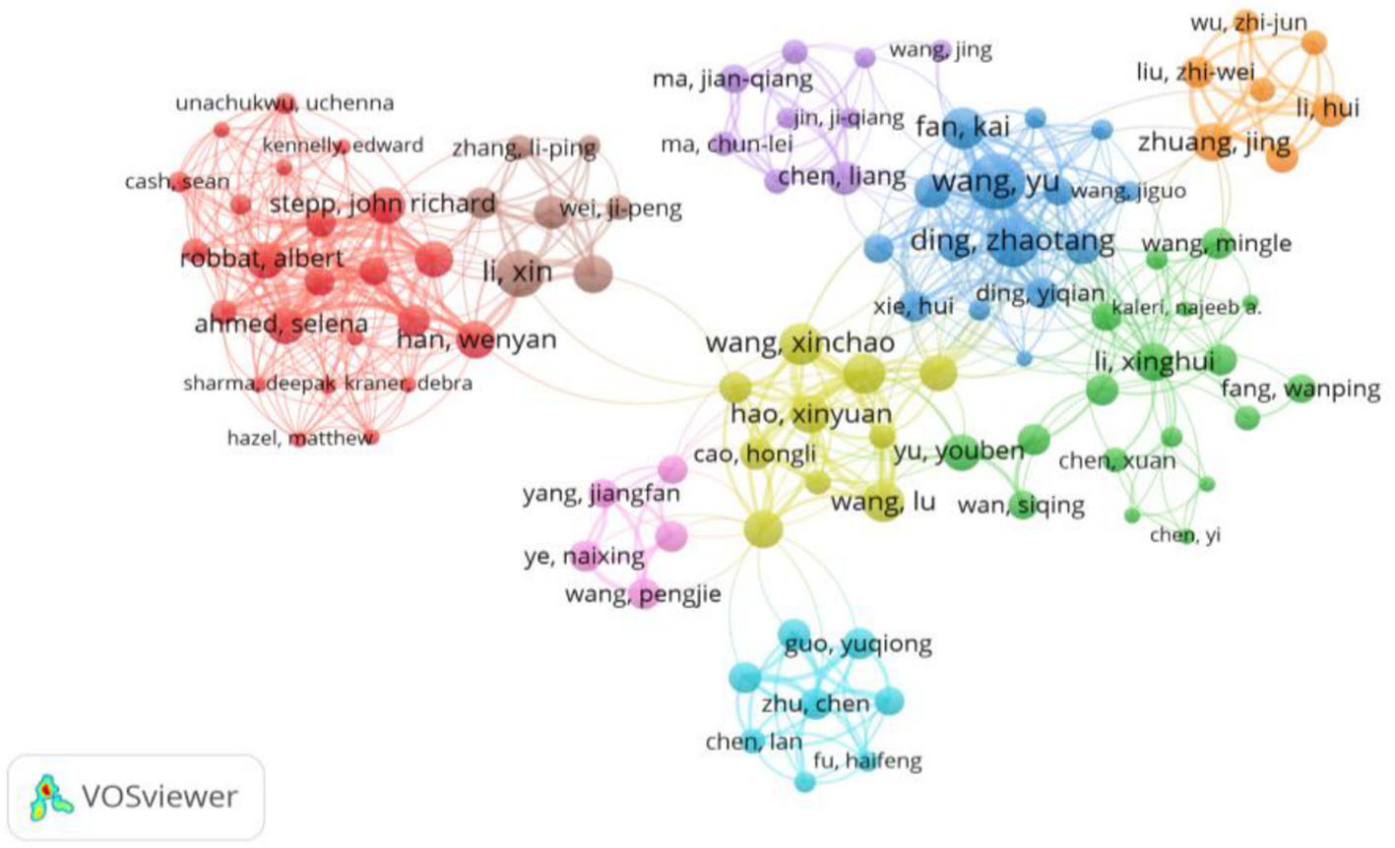
Collaborative network of influential authors in climate change research on tea cultivation: This VOSviewer visualization maps the co-authorship network of prominent researchers contributing to studies on climate change impacts on tea cultivation. Each node represents an author, with node size reflecting the author’s research output or influence within this domain. The lines connecting nodes indicate co-authorship relationships, and the thickness of these lines represents the strength or frequency of collaboration between authors. Color-coded clusters identify groups of closely associated researchers, highlighting collaborative sub-networks within the field. Notable authors such as Wang Yu, Ding Zhaotang, and Li Xin appear as central figures, denoting their significant roles in advancing research and fostering partnerships in this area. This network offers a comprehensive view of the collaborative landscape, showcasing the interconnectedness of leading scholars and the structure of knowledge-sharing within climate and tea cultivation studies. Created with VOSviewer.

**Figure 9:**
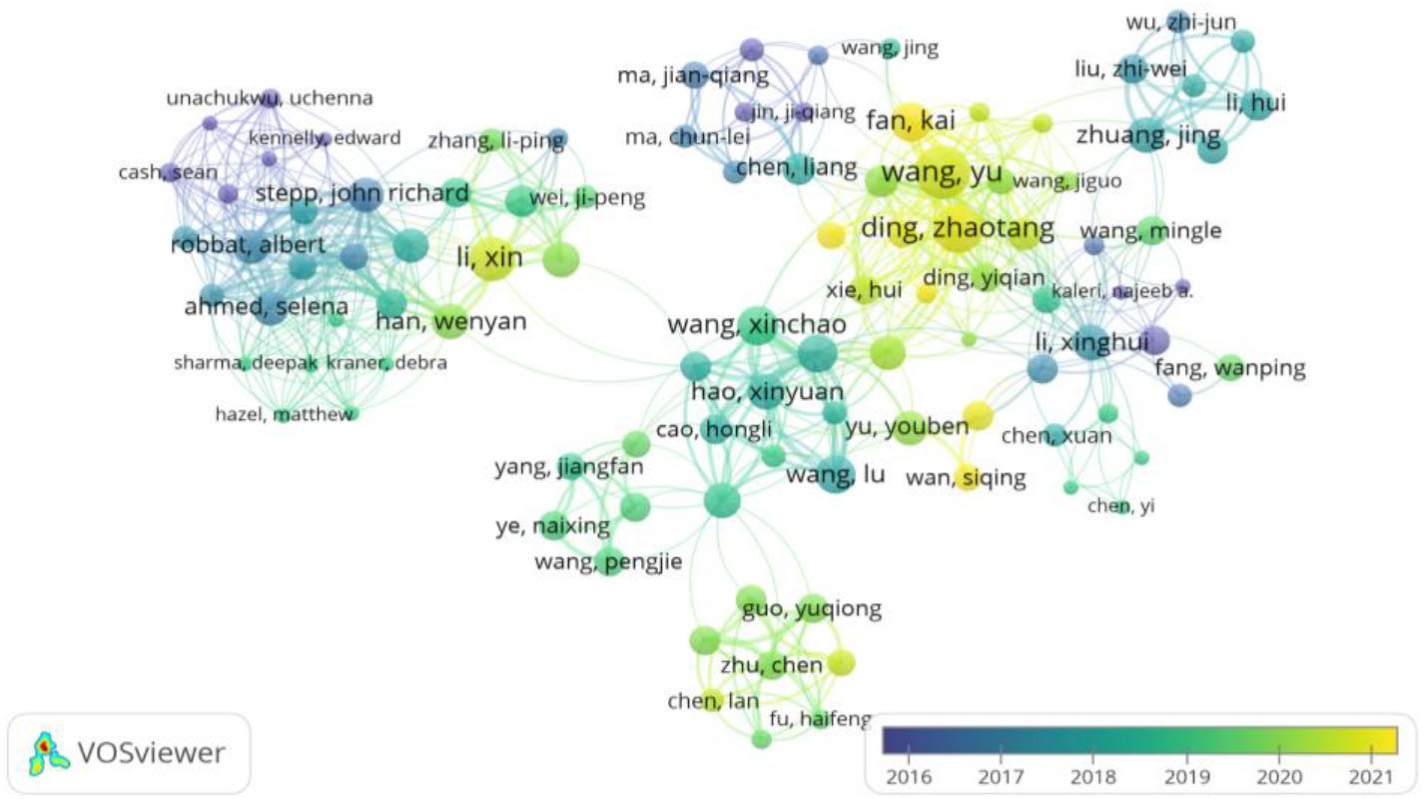
Temporal evolution of collaborative network among influential authors in climate change research on tea cultivation: This VOSviewer visualization illustrates the temporal dynamics of co-authorship networks among prominent researchers in climate change impacts on tea cultivation, covering the period from 2016 to 2021. Each node represents an author, with node size reflecting the author’s influence or publication output, while color shading indicates the timing of their contributions, as per the color gradient. The connections between nodes signify co-authorship, with thicker lines denoting stronger or more frequent collaborations. The color gradient provides insight into research clusters’ formation and evolution, showcasing long-standing and newly emerging partnerships. For instance, some collaborative groups have intensified their activities recently, as indicated by the lighter shades. This temporal view of the network highlights the dynamic nature of scholarly collaboration, shedding light on how influential researchers and their teams adapt and respond to emerging challenges in climate change and tea cultivation research. Created with VOSviewer.

**Figures 8** and **9**, generated through *VOSviewer* visualizations, offer an in-depth view of the collaborative networks among authors engaged in research on the impacts of climate change on tea cultivation. These visualizations are based on co-authorship analysis, with clusters representing groups of researchers who frequently work together within this specialized field. By examining these networks, we can identify key contributors, collaborative patterns, and the structure of research partnerships that drive advancements in this area.

**Figure 8** offers a general overview of these co-authorship networks. Each color-coded cluster represents a distinct group of closely connected authors, with prominent researchers occupying central positions within their clusters. For instance, authors like *Wang Yu, Ding Zhaotang*, and *Li Xin* emerge as central figures, indicating their influential roles and strong collaborative networks. These authors act as focal points within their respective clusters, likely due to their extensive publication records, involvement in multiple studies, or leadership in research projects on tea and climate change. The well-connected authors suggest that specific individuals or groups may serve as hubs of expertise, facilitating knowledge-sharing and significantly contributing to the development of the field.

The visualization in **Figure 8** also illustrates how researchers are organized into various clusters, each representing a group of co-authors who frequently collaborate on studies within this domain. These clusters provide insight into how research on climate change impacts tea cultivation is progressing, revealing that certain groups may focus on specific aspects of the field, such as genetic adaptations, physiological responses, or environmental stressors affecting tea plants.

**Figure 9** adds a temporal dimension to the co-authorship networks, displaying the evolution of collaborations over time from 2016 to 2021. The color gradient, ranging from darker shades (earlier years) to brighter shades (more recent years), indicates the development and shifts in research partnerships. This temporal view highlights how collaborative relationships within the tea and climate change research community have evolved, showing the growth of specific networks and the emergence of new collaborative partnerships.

For example, *Wang Yu and Ding Zhaotang* appear to have intensified their collaborative efforts in recent years, as indicated by the brighter shades surrounding these authors. This suggests that these researchers have been actively involved in recent studies, contributing to the latest developments in this field. The visualization also highlights newly established connections, reflecting a dynamic research environment where emerging authors and new collaborations are shaping the landscape of tea and climate change research.

Together, **Figures 8** and **9** offer a comprehensive perspective on the collaborative landscape in climate change research on tea cultivation. These visualizations provide valuable insights into the key contributors, the structure of research partnerships, and the temporal evolution of collaborations, helping identify influential researchers and emerging collaborations that will drive further progress in addressing climate change impacts on tea cultivation.

### 3.8 Impacts of climate variables on tea cultivation across regions and adaptive strategies for resilience

The analysis reveals that the primary climate variables affecting tea cultivation include temperature fluctuations, rainfall variability, drought stress, and humidity. These factors significantly impact tea quality and yield across regions, with distinct patterns based on geography. For example, drought-induced stress is a predominant challenge in countries like India and Kenya, leading to reduced chlorophyll and catechin levels and lower biomass production (Cheruiyot et al., 2007; Singh et al., 2009). Similarly, extreme temperature fluctuations and water scarcity in China result in oxidative stress and reduced bioactive compound concentrations in tea leaves, such as catechins and theanine (Wang et al., 2016; Li et al., 2019). In high-altitude regions like Sri Lanka, cooler temperatures improve tea quality but reduce yields due to lower polyphenol content (Jayasinghe and Kumar, 2019).

**Supplementary Table 1** provides a comprehensive summary of the primary climate variables affecting tea cultivation across countries, along with the adaptation strategies employed to mitigate these challenges. For example, temperature and drought stress are dominant factors in subtropical regions such as India and Kenya, significantly affecting catechin levels and leaf growth (Cheruiyot et al., 2007; Singh et al., 2009). In tropical regions like China’s Yunnan Province, seasonal precipitation variability is particularly impactful, influencing tea quality and metabolite concentrations, especially during the monsoon season (Ahmed et al., 2014).

Adaptation strategies to mitigate the impacts of climate change on tea cultivation encompass a wide range of agronomic practices, biochemical interventions, and molecular approaches. Traditional techniques such as agroforestry and soil conservation remain crucial in tropical regions like Sri Lanka, where shade trees buffer temperature extremes and reduce moisture variability. These practices offer cost-effective solutions for smallholder farmers, with low implementation costs and the ability to complement existing farming systems (Wijeratne et al., 2007).

Adaptation strategies to mitigate the impacts of climate change on tea cultivation encompass a wide range of agronomic practices, biochemical interventions, and molecular approaches. Traditional techniques such as agroforestry and soil conservation remain crucial in tropical regions like Sri Lanka, where shade trees buffer temperature extremes and reduce moisture variability. These practices offer cost-effective solutions for smallholder farmers, with low implementation costs and the ability to complement existing farming systems (Upadhyaya et al., 2011; Li et al., 2019). However, the application of these biochemical solutions poses challenges for small-scale farmers due to the costs of chemical inputs and the technical expertise required for their precise application. Cost-benefit analyses of these interventions suggest that, while they can substantially improve yields, their practicality depends on subsidies, farmer training, and access to affordable alternatives.

Molecular tools such as RNA-Seq, miRNA profiling, and proteomics have identified key genes involved in stress tolerance, offering significant potential for breeding resilient tea cultivars (Liu et al., 2016; Gu et al., 2020). However, the high costs of genomic technologies and their reliance on advanced infrastructure limit their widespread adoption in developing regions. For smallholder farmers, the benefits of these molecular approaches can be realized only through collaborative efforts between research institutions and policymakers to subsidize costs, simplify technology transfer, and ensure scalability. Integrating molecular breeding programs with traditional agronomic practices, such as agroforestry, can help maximize the impact of these innovations while addressing the financial and technical barriers faced by farmers.

To enhance the practicality of these adaptation strategies, it is essential to integrate modern molecular and biochemical solutions with traditional agricultural practices. Case studies from regions like Yunnan, China, demonstrate that pairing agroforestry with low-cost biochemical applications, such as melatonin sprays, effectively mitigates drought stress while improving soil quality and crop resilience. Similarly, simulation models and climate suitability tools can guide farmers in selecting appropriate adaptation measures tailored to their regional conditions, further enhancing operational efficiency and sustainability.

Policymakers and researchers must prioritize developing region-specific adaptation strategies that account for financial constraints and technical challenges. By aligning scientific innovations with farmer-oriented applications, these strategies can ensure the long-term sustainability of tea cultivation under climate change.

#### 3.8.1 Low temperatures as a critical climate variable

Low temperatures during the spring present a significant challenge to tea cultivation, especially in high-altitude regions like the Middle and Lower Reaches of the Yangtze River in China. Spring frost damage (SFD) is a major concern, leading to restricted production and reduced quality of spring tea. Physiological and biochemical changes induced by cold stress include oxidative stress, increased ROS accumulation, and decreased levels of chlorophyll and carotenoids, all of which negatively affect photosynthesis and tea quality (Li X. et al., 2018; Wang et al., 2021). Young tea leaves are particularly vulnerable, exhibiting higher damage and reduced photosynthetic efficiency compared to mature leaves due to differences in gene expression related to cell membrane stability and ROS detoxification (Li N. et al., 2018).

Adaptive strategies include the application of exogenous compounds like 5-aminolevulinic acid (ALA) and melatonin, which have been shown to enhance cold tolerance by improving antioxidant defenses and modulating stress-related pathways (Hao et al., 2018; Yan et al., 2023). Combined with agronomic interventions like shading and site selection, these strategies can help mitigate the adverse effects of spring frost damage.

### 3.9 Emerging trends, knowledge gaps, and research directions in tea cultivation under climate stress

#### 3.9.1 Emerging trends in tea cultivation

Emerging technologies and approaches have brought significant advancements in tea cultivation to mitigate climate impacts. A key trend is the application of omics tools, such as transcriptomics, proteomics, and miRNA profiling, which have helped identify regulatory networks and pathways responsible for stress resilience. For example, RNA-Seq has revealed extensive transcriptional changes during stress and recovery phases in tea plants, shedding light on genes related to drought and cold stress responses (Liu et al., 2016; Zheng et al., 2016). Another emerging trend is the use of biochemical interventions, including exogenous melatonin, ABA, and CaCl₂. These compounds have the potential to improve antioxidant defense systems, enhance drought tolerance, and support post-stress recovery in controlled environments (Upadhyaya et al., 2011; Li et al., 2019). Crop modeling tools, such as MaxEnt species distribution models, are increasingly used to predict climate suitability and guide cultivation practices under future scenarios (Jayasinghe and Kumar, 2019).

**Supplementary Table 2** summarizes these trends and their impacts on tea cultivation. For example, polyphenols have been identified as biochemical markers for drought tolerance, enabling breeders to select resilient cultivars (Cheruiyot et al., 2007). Additionally, transcriptomics and metabolomics studies have been integrated to better understand tea plant responses to multiple stress factors (Gai et al., 2020).

#### 3.9.2 Knowledge gaps in tea cultivation

While advancements have been significant, several knowledge gaps hinder the translation of research findings into practical applications. Many biochemical and molecular innovations lack field-based validation. For example, the long-term effects of exogenous compounds like melatonin on plant yield and stress response remain insufficiently studied (Li et al., 2019). Similarly, the impact of elevated CO₂ concentrations on tea secondary metabolites is not well understood, with contradictory findings highlighting the need for standardized protocols and long-term assessments (Li et al., 2017). Furthermore, the scalability of technologies such as foliar applications of ABA and CaCl₂ requires further exploration to ensure their affordability and feasibility for smallholder farmers.

#### 3.9.3 Future research directions

Future research should address these knowledge gaps by expanding trials to diverse agro-ecological regions and integrating findings into real-world farming systems. For instance, proteomic insights into drought-induced changes in lignin and flavonoid biosynthesis (Gu et al., 2020) must be validated in field conditions across varying climates. Furthermore, integrating climate models with socioeconomic analyses will help develop equitable adaptation strategies (Wijeratne et al., 2007). These directions will ensure that molecular and biochemical innovations contribute effectively to improving resilience in tea cultivation.

## 4 Discussion

### 4.1 Trends in research on climate change impacts on tea cultivation

The upward trajectory in research output, as observed in Section 3.1, highlights the growing academic focus on the effects of climate change on tea cultivation, particularly after 2014. This trend mirrors the global recognition of climate change as a significant challenge for agriculture. The surge in publications post-2020 reflects intensified climate stresses in key tea-growing regions and a global push toward sustainable agricultural practices (Wijeratne et al., 2007; Zhou et al., 2014). However, the earlier period (2004–2013) saw limited attention, suggesting that the specific vulnerabilities of tea crops to climate change were not yet recognized as a priority. This shift in research focus underscores the need for continued efforts to address the gaps identified in earlier studies and to support regions that are only now experiencing severe climate impacts (Ahmed et al., 2014).

### 4.2 Most influential authors, sources, and countries

Key contributors, such as Kumar Sanjay and Ding Zhaotang, have driven research into various subfields, including plant physiology, molecular genetics, and environmental resilience (Singh et al., 2008; Wang et al., 2016). Journals like Frontiers in Plant Science have become central platforms for disseminating findings, reinforcing the interdisciplinary nature of this field (Zheng et al., 2016). Geographically, China and India lead in research output, reflecting their status as major tea producers (Cheruiyot et al., 2007; Jayasinghe and Kumar, 2019). However, contributions from African and Southeast Asian nations, where tea is equally critical, remain limited. This imbalance underscores the need for expanded research collaborations and increased funding in underrepresented regions to address the specific challenges faced by their tea industries (Zou et al., 2014).

### 4.3 Most influential documents on climate change impacts on tea cultivation

The analysis of influential documents in Section 3.4 underscores key findings that have shaped the field. For instance, Singh et al. (2008) demonstrated how environmental stressors influence catechin biosynthesis, linking biochemical responses to climate resilience. Similarly (Li et al., 2019), highlighted the role of melatonin in enhancing tea plant tolerance to multiple stresses, providing practical adaptation strategies. Studies like Jayasinghe and Kumar (2019) advanced our understanding of climate suitability in Sri Lanka, offering a regional perspective essential for tailoring mitigation strategies. While these foundational works have provided significant insights, the limited focus on interactive stressors, such as the combination of drought and heat, indicates a need for more integrative studies that address the complex, real-world challenges faced by tea farmers (Wang et al., 2016; Zheng et al., 2016).

### 4.4 Disciplinary distribution of research

The disciplinary distribution identified in Section 3.5 reflects a strong emphasis on Agricultural and Biological Sciences, which account for 36% of the research. This aligns with the need to explore physiological and biochemical adaptations in tea plants to climate stressors (Hernández et al., 2006; Upadhyaya et al., 2011). However, the underrepresentation of Social Sciences and Environmental Policy Research highlights a gap in addressing the socioeconomic and governance dimensions of climate change’s impact on tea farming (Wijeratne et al., 2007). Expanding research in these areas could bridge the gap between scientific findings and practical, scalable interventions for vulnerable farming communities, ensuring that climate adaptation strategies are both effective and sustainable.

### 4.5 Primary research themes

The keyword analysis in Section 3.6 identifies drought tolerance, antioxidant responses, and molecular adaptations as dominant themes in the research on climate change impacts on tea cultivation. Studies such as those by (Zhou et al., 2014) and (Wang et al., 2016) have advanced our understanding of how specific genes and metabolic pathways enable tea plants to withstand environmental stressors. Emerging themes, such as water management and photosynthesis under elevated CO₂, reflect an increasing focus on sustainability and quality improvement in response to climate change (Ahmed et al., 2014; Li et al., 2017). However, the limited focus on farmer-driven adaptation strategies suggests a gap between academic research and practical field applications. Future studies should prioritize generating actionable insights that are aligned with the needs of smallholder farmers, ensuring that research outcomes are applicable to real-world farming conditions (Guo et al., 2017).

Cold stress has emerged as a significant focus area in climate change research on tea cultivation, as highlighted in the keyword co-occurrence analysis (**Figure 7**). The strong associations of “cold stress” with terms like “abiotic stress,” “expression profile,” and “transcriptome” underscore the growing emphasis on understanding tea plants’ responses to low-temperature challenges. Research in this area focuses on key physiological and molecular mechanisms, such as cold acclimation processes, antioxidant activities, and transcriptomic changes, which contribute to the plant’s ability to withstand cold stress. These findings not only provide insights into the adaptive capacity of tea plants but also inform the development of strategies to enhance their resilience under changing climatic conditions.

### 4.6 Collaborative networks

As analyzed in Section 3.7, collaborative networks play a critical role in advancing research on climate change impacts on tea cultivation. Prominent authors like Wang Yu and Ding Zhaotang act as central figures in co-authorship networks, facilitating interdisciplinary approaches to complex problems (Singh et al., 2008; Zheng et al., 2016). However, the segmentation of these networks suggests limited integration of global expertise, with collaborations largely concentrated within specific regions or disciplines. Encouraging broader cross-regional and interdisciplinary partnerships could enhance knowledge-sharing and foster innovation, particularly in regions with limited research capacity (Zou et al., 2014; Jayasinghe and Kumar, 2019). Expanding these networks would help address the interconnected challenges faced by tea industries in diverse regions.

### 4.7 Synthesizing regional insights and adaptation strategies to mitigate climate impacts on tea cultivation

The regional variations in climate impacts on tea cultivation highlight the complex interplay between environmental factors and plant physiology. Drought stress emerges as a critical variable in low-altitude regions, exacerbating oxidative damage and reducing leaf quality. Conversely, high-altitude environments offer a natural advantage by mitigating temperature-related stress, but they face challenges in maintaining yields due to slower growth rates and altered metabolite profiles (Han et al., 2017). Seasonal precipitation shifts further complicate tea production, particularly in subtropical areas like Yunnan, China, where the onset of the monsoon affects the synthesis of secondary metabolites (Ahmed et al., 2014).

While traditional agronomic practices offer a solid foundation for climate adaptation, integrating modern technologies is essential for ensuring long-term sustainability. Biochemical applications, such as fulvic acid and polyphenols, improve stress tolerance and enhance tea quality by regulating antioxidant and flavonoid pathways (Zhou et al., 2014; Sun et al., 2020). However, the scalability of these interventions remains a challenge, especially for smallholder farmers in developing regions. Similarly, molecular insights from transcriptomic and proteomic studies need to be translated into field applications to validate their efficacy under natural conditions (Guo et al., 2017; Wang et al., 2018).

Emerging trends such as precision agriculture and the development of climate-resilient cultivars offer promising avenues for the future of tea cultivation. However, significant gaps persist particularly the limited understanding of long-term climatic impacts on tea quality and the lack of holistic models that integrate socioeconomic factors. Addressing these gaps requires multidisciplinary approaches that combine molecular, agronomic, and economic perspectives to ensure both productivity and sustainability in the face of climate change.

The impact of low temperatures during spring, especially in high-altitude regions, adds another layer of complexity to climate adaptation strategies for tea cultivation. Spring frost damage (SFD) not only reduces yields but also affects tea quality by altering chlorophyll, carotenoid, and metabolite concentrations (Wang et al., 2021; Yang et al., 2021). The impact of low temperatures during spring, especially in high-altitude regions, adds another layer of complexity to climate adaptation strategies for tea cultivation. Spring frost damage (SFD) not only reduces yields but also affects tea quality by altering chlorophyll, carotenoid, and metabolite concentrations (Li N. et al., 2018).

Mitigating these challenges requires a multifaceted approach. Biochemical applications, such as exogenous melatonin and 5-aminolevulinic acid (ALA), have shown efficacy in enhancing cold tolerance by activating antioxidant pathways and regulating stress-resistance genes (Hao et al., 2018). However, translating these laboratory findings into scalable field practices remains a challenge. Future research should focus on optimizing these interventions for smallholder farmers while exploring traditional practices, such as agroforestry and shading, to provide cost-effective solutions for managing low-temperature stress. Additionally, breeding programs targeting enhanced cold tolerance through insights from transcriptomics and proteomics could play a pivotal role in building resilience to low-temperature stress.

### 4.8 Integrating innovations and addressing challenges for sustainable tea cultivation in a changing climate

#### 4.8.1 Synthesis of emerging trends

Emerging trends in tea cultivation highlight the importance of integrating traditional agricultural practices with modern molecular and information technologies to enhance crop resilience. Omics technologies, such as RNA-Seq, have revealed extensive regulatory networks that govern stress adaptation, identifying critical targets for breeding stress-resilient tea cultivars (Liu et al., 2016; Zheng et al., 2016). Genomics-assisted breeding techniques, including the use of transgenes, have demonstrated success in creating elite genotypes capable of withstanding drought and temperature extremes, providing a robust foundation for future innovations (Seth et al., 2021; Ramakrishnan et al., 2023). Biochemical interventions, such as fulvic acid and polyphenols, have shown significant potential in mitigating abiotic stresses while simultaneously improving tea quality. These modern approaches complement traditional practices, such as agroforestry and soil conservation, which can serve both as adaptation and mitigation strategies. Implementing agroforestry—such as maintaining shade trees in tea farms—helps reduce the climate footprint and enhances the resilience of tea systems. Furthermore, advancements in information technology, including climate suitability modeling and high-resolution simulation models, enable more precise planning for tea cultivation under current and future climatic scenarios. These tools help mitigate risks and optimize productivity, making tea farming more resilient to climate variability (Jayasinghe and Kumar, 2019; Muoki et al., 2020).

#### 4.8.2 Addressing knowledge gaps

Despite these advancements, several knowledge gaps and practical challenges hinder the effective implementation of these strategies. For example, while the application of abscisic acid (ABA) has shown promise in controlled environments for enhancing lipid and flavonoid metabolism under drought stress, its long-term effects in field conditions remain unclear (Gai et al., 2020). Similarly, molecular insights from miRNA profiling and transcriptomics have identified key regulatory pathways for stress tolerance; however, translating these findings into breeding programs for large-scale adoption still requires substantial effort (Guo et al., 2017).

Traditional practices, such as agroforestry, must be integrated with modern technologies to maximize their potential. For instance, combining agroforestry with advanced soil management techniques—such as biochar and controlled-release fertilizers—can improve nitrogen use efficiency, reduce greenhouse gas emissions, and support sustainable tea cultivation (Wang et al., 2020). Moreover, precision agriculture and climate modeling tools require further refinement to address socioeconomic factors and ensure these technologies are accessible to resource-limited farmers.

#### 4.8.3 Implications for future research

Future research must adopt an interdisciplinary approach that combines molecular, agronomic, and economic perspectives to address the multifaceted challenges posed by climate change. Long-term field trials are essential to validate laboratory findings, such as the efficacy of heat shock proteins (HSPs) and other molecular targets, under diverse growing conditions (Seth et al., 2021). Genomics-assisted breeding programs should prioritize traits that enhance resistance to both biotic and abiotic stresses, such as drought and heat tolerance, while also considering the economic viability of farmers (Ramakrishnan et al., 2023).

Advanced information technologies, such as MaxEnt climate suitability modeling and simulation tools, should be integrated with socioeconomic analyses to ensure that adaptation strategies are both equitable and effective (Wijeratne et al., 2007) (Jayasinghe and Kumar, 2019). For example, incorporating these tools into farmer education programs could help smallholder farmers plan and adapt to future climatic conditions. Additionally, expanding agroforestry research to include its synergistic effects with molecular interventions could provide innovative pathways for sustainable tea cultivation.

By bridging these knowledge gaps and combining traditional practices with cutting-edge innovations, the tea industry can develop robust strategies to enhance productivity and resilience in the face of a changing climate.

## 5 Conclusions

This study presents a comprehensive bibliometric and content analysis of research on the impacts of climate change on tea cultivation, synthesizing key trends, influential contributions, and critical knowledge gaps. Over the past two decades, there has been a growing focus on understanding the physiological, biochemical, and molecular responses of tea plants to climate stressors, including drought, temperature extremes, low temperatures, and elevated CO₂ levels. Influential studies have elucidated resilience mechanisms, such as antioxidant defense systems, secondary metabolite regulation, and genomic adaptations, which are vital for developing targeted adaptation strategies.

Our review of highly cited studies and thematic clusters identified the primary climate variables impacting tea cultivation, highlighted innovative adaptation strategies, and proposed future research priorities. Despite these advancements, challenges persist in addressing the multifaceted impacts of climate change on tea cultivation. These challenges include limited research on the synergistic effects of multiple climate stressors, insufficient field validation of molecular and genomic findings, and inadequate consideration of socioeconomic factors influencing climate adaptation strategies. Addressing these challenges requires integrating molecular technologies, such as genomics-assisted breeding and transcriptomics, with advanced information tools like climate suitability modeling and simulation. Moreover, expanding interdisciplinary research efforts to include socioeconomic and environmental dimensions is essential for developing scalable, farmer-oriented, and region-specific solutions.

Future research must prioritize underrepresented regions, such as Africa and Southeast Asia, where tea cultivation is economically significant and culturally embedded. Strengthening international collaborations and aligning scientific innovations with practical applications for smallholder farmers can bridge the gap between research and implementation. By addressing these gaps, the tea industry can enhance its resilience to climate change, ensure sustainable production practices, and safeguard the livelihoods of millions who depend on this vital crop.

## 6 Limitations

While this study provides valuable insights into the impacts of climate change on tea cultivation, several limitations must be acknowledged and addressed in future research.

First, the analysis is heavily reliant on publications indexed in specific databases, such as Scopus, which may exclude relevant studies published in regional or less widely indexed journals. This limitation likely underrepresents research contributions from regions such as Africa and Southeast Asia, where tea cultivation is both economically significant and deeply integrated into local agricultural practices. Research from these regions often employs unique methodologies and perspectives that remain underrepresented in global analyses. Future efforts should expand the scope to include regional databases or collaborate directly with local researchers to ensure a more comprehensive representation of global research.

Second, the bibliometric approach primarily emphasizes citation metrics to evaluate research impact. While this method effectively identifies highly cited studies, it tends to favor older publications over more recent, innovative research that may not have had sufficient time to accumulate citations. For instance, recent advancements in genomic technologies or precision agriculture might not emerge as influential documents despite their transformative potential. Future analyses should incorporate alternative metrics, such as altmetrics or expert evaluations, to more effectively capture the value of emerging research.

Third, this study predominantly focuses on academic research, with limited integration of industry reports, farmer-centric studies, or grey literature. These sources are critical for understanding real-world challenges and the practical applications of adaptation strategies. For example, industry reports provide timely data on economic trends and technological adoption, while farmer-centric studies can highlight adaptation strategies that have proven successful at the local level. Incorporating such diverse perspectives would bridge the gap between academic findings and practical implementation.

Fourth, the study does not systematically evaluate the methodological rigor or quality of the individual studies included in the analysis. This omission may limit the reliability of insights drawn from certain sources. A systematic critical appraisal of research methodologies—considering factors such as study design, sample size, and statistical validity—would enhance the robustness of future syntheses and ensure the applicability of findings across diverse agroecological and socioeconomic contexts.

Finally, while this study identifies key knowledge gaps and research directions, it does not fully address the dynamic interplay between socioeconomic and environmental factors that influence tea cultivation. Expanding future analyses to include integrated models that account for these variables will provide a more holistic understanding of climate change impacts.

Despite these limitations, this study represents a foundational step in synthesizing and advancing research on the impacts of climate change on tea cultivation. It provides a comprehensive roadmap for addressing critical gaps and fostering sustainable agricultural practices to mitigate the challenges posed by a changing global climate.

## References

[B1] Ahmed S. Griffin T. S. Kraner D. Schaffner M. K. Sharma D. Hazel M.. (2019). Environmental factors variably impact tea secondary metabolites in the context of climate change. Front. Plant Sci. 10. doi: 10.3389/fpls.2019.00939 PMC6702324 31475018

[B2] Ahmed S. Stepp J. R. Orians C. Griffin T. Matyas C. Robbat A.. (2014). Effects of extreme climate events on tea (Camellia sinensis) functional quality validate indigenous farmer knowledge and sensory preferences in Tropical China. PloS One 9. doi: 10.1371/journal.pone.0109126 PMC4186830 25286362

[B3] Aria M. Cuccurullo C. (2017). bibliometrix: An R-tool for comprehensive science mapping analysis. J. Informetrics 11, 959–975. doi: 10.1016/j.joi.2017.08.007

[B4] Ashraf M. A. Murtaza N. Brown J. K. Yu N. (2023). In silico identification of apple genome-encoded microRNA target binding sites potentially targeting the ACLSV’. Basel, Switzerland: MDPI (Horticultura). doi: 10.20944/preprints202305.0517.v1

[B5] Chen Y. Li Y. Shen C. Xiao L. (2023). Topics and trends in fresh tea (Camellia sinensis) leaf research: A comprehensive bibliometric study. Front. Plant Sci. 14. doi: 10.3389/fpls.2023.1092511 PMC10118041 37089662

[B6] Cheruiyot E. K. Mumera L. M. Ng’etich W. K. Hassanali A. Wachira F. (2007). Polyphenols as potential indicators for drought tolerance in tea (Camellia sinensis L.). Bioscience Biotechnol. Biochem. 71, 2190–2197. doi: 10.1271/bbb.70156 17827703

[B7] Elisha I. L. Viljoen A. (2021). Trends in Rooibos Tea (Aspalathus linearis) research (1994ndash;2018): A scientometric assessment. South Afr. J. Botany 137 pp, 159–170. doi: 10.1016/j.sajb.2020.10.004

[B8] Fadhlina A. Alias N. F. A. Sheikh H. I. Zakaria N. H. Majid F. A. A. Hairani M. A. S.. (2023). Role of herbal tea (Camellia sinensis L. Kuntze, Zingiber officinale Roscoe and Morinda citrifolia L.) in lowering cholesterol level: A review and bibliometric analysis. J. Agric. Food Res. 13, 100649. doi: 10.1016/j.jafr.2023.100649

[B9] Gai Z. Wang Y. Ding Y. Qian W. Qiu C. Xie H.. (2020). Exogenous abscisic acid induces the lipid and flavonoid metabolism of tea plants under drought stress. Sci. Rep. 10. doi: 10.1038/s41598-020-69080-1 PMC7378251 32704005

[B10] Gu H. Zhang X. Lam S. K. Yu Y. Van Grinsven H. J. M. Zhang S.. (2020). Drought stress triggers proteomic changes involving lignin, flavonoids and fatty acids in tea plants. Sci. Rep. 10. doi: 10.1038/s41598-020-72596-1 PMC7511325 32968186

[B11] Gu B. Zhang X. Lam S. K. Yu Y. Van Grinsven H. J. M. Zhang S.. (2023). Cost-effective mitigation of nitrogen pollution from global croplands. Nature 613, 77–84. doi: 10.1038/s41586-022-05481-8 36600068 PMC9842502

[B12] Guo Y. Zhao S. Zhu C. Chang X. Yue C. Wang Z.. (2017). Identification of drought-responsive miRNAs and physiological characterization of tea plant (Camellia sinensis L.) under drought stress. BMC Plant Biol. 17. doi: 10.1186/s12870-017-1172-6 PMC5696764 29157225

[B13] Han W.-Y. Huang J.-G. Li X. Li Z.-X. Ahammed G. J. Yan P.. (2017). Altitudinal effects on the quality of green tea in east China: a climate change perspective. Eur. Food Res. Technol. 243, 323–330. doi: 10.1007/s00217-016-2746-5

[B14] Hao X. Wang B. Wang L. Zeng J.-M. Yang Y. Wang X. (2018). Comprehensive transcriptome analysis reveals common and specific genes and pathways involved in cold acclimation and cold stress in tea plant leaves. Scientia Hortic. 240, 354–368. doi: 10.1016/J.SCIENTA.2018.06.008

[B15] Hernández I. Alegre L. Munné-Bosch S. (2006). Enhanced oxidation of flavan-3-ols and proanthocyanidin accumulation in water-stressed tea plants. Phytochemistry 67, 1120–1126. doi: 10.1016/j.phytochem.2006.04.002 16712885

[B16] Jayasinghe S. L. Kumar L. (2019). Modeling the climate suitability of tea [Camellia sinensis(L.) O. Kuntze] in Sri Lanka in response to current and future climate change scenarios. Agric. For. Meteorol. 272-273, 102–117. doi: 10.1016/j.agrformet.2019.03.025

[B17] Li X. Zhang L. Ahammed G. J. Li Z.-X. Wei J.-P. Shen C.. (2017). Stimulation in primary and secondary metabolism by elevated carbon dioxide alters green tea quality in Camellia sinensis L. Sci. Rep. 7. doi: 10.1038/s41598-017-08465-1 PMC5554289 28801632

[B18] Li N. Yue C. Cao H. Qian W. Hao X. Wang Y.. (2018). Transcriptome sequencing dissection of the mechanisms underlying differential cold sensitivity in young and mature leaves of the tea plant (Camellia sinensis). J. Plant Physiol. 224–225, 144–155. doi: 10.1016/j.jplph.2018.03.017 29642051

[B19] Li X. Wei J.-P. Scott E. Liu J.-W. Guo S. Li Y.. (2018). Exogenous melatonin alleviates cold stress by promoting antioxidant defense and redox homeostasis in camellia sinensis L. Molecules : A J. Synthetic Chem. Natural Product Chem. 23, 165. doi: 10.3390/molecules23010165 PMC6017414 29342935

[B20] Li J. Yang Y. Sun K. Chen Y. Chen X. Li X. (2019). Exogenous melatonin enhances cold, salt and drought stress tolerance by improving antioxidant defense in tea plant (Camellia sinensis (L.) O. Kuntze). Molecules 24. doi: 10.3390/molecules24091826 PMC6539935 31083611

[B21] Li Y. Chen Y. Chen J. Shen C. (2023). Flavonoid metabolites in tea plant (Camellia sinensis) stress response: Insights from bibliometric analysis. Plant Physiol. Biochem. 202, 107934. doi: 10.1016/j.plaphy.2023.107934 37572493

[B22] Liu S.-C. Jin J.-Q. Ma J.-Q. Yao M.-Z. Ma C.-L. Li C.-F.. (2016). Transcriptomic analysis of tea plant responding to drought stress and recovery. PloS One 11. doi: 10.1371/journal.pone.0147306 PMC4720391 26788738

[B23] Liu S. Fan B. Li X. Sun G. (2024). Global hotspots and trends in tea anti-obesity research: a bibliometric analysis from 2004 to 2024. Front. Nutr. 11. doi: 10.3389/fnut.2024.1496582 PMC11598529 39606571

[B24] Muoki C. Maritim T. Oluoch W. A. Kamunya S. Bore J. (2020). Combating climate change in the Kenyan tea industry. Front. Plant Sci. 11. doi: 10.3389/fpls.2020.00339 PMC7109314 32269583

[B25] Page M. J. McKenzie J. E. Bossuyt P. M. Boutron I. Hoffmann T. C. Mulrow C. D.. (2021). The PRISMA 2020 statement: an updated guideline for reporting systematic reviews. BMJ 372, n71. doi: 10.1136/bmj.n71 33782057 PMC8005924

[B26] Ramakrishnan M. Sudhama V. Rajanna L. (2023). A review on the genome-based approaches for the development of stress and climate resilient tea crops. Plant Sci. Today. 9 (sp3), 105–109. doi: 10.14719/pst.1758

[B27] Ramírez-Gottfried R. I. Preciado-Rangel P. Carrillo M. G. García A. B. González-Rodríguez G. Espinosa-Palomeque B. (2023). Compost tea as organic fertilizer and plant disease control: bibliometric analysis. Agronomy 13, 2340. doi: 10.3390/agronomy13092340

[B28] Sahu N. Nayan R. Panda A. Varun A. Kesharwani R. Das P. (2025). Impact of changes in rainfall and temperature on production of Darjeeling Tea in India. Atmosphere. 16, 1. doi: 10.3390/atmos16010001

[B29] Seth R. Maritim T. Parmar R. Sharma R. (2021). Underpinning the molecular programming attributing heat stress associated thermotolerance in tea (Camellia sinensis (L.) O. Kuntze). Horticulture Res. 8. doi: 10.1038/s41438-021-00532-z PMC8087774 33931616

[B30] Singh K. Rani A. Kumar S. Sood P. Mahajan M. Yadav S. K.. (2008). An early gene of the flavonoid pathway, flavanone 3-hydroxylase, exhibits a positive relationship with the concentration of catechins in tea (Camellia sinensis). Tree Physiol. 28, 1349–1356. doi: 10.1093/treephys/28.9.1349 18595847

[B31] Singh K. Kumar S. Rani A. Gulati A. Ahuja P. S. (2009). Phenylalanine ammonia-lyase (PAL) and cinnamate 4-hydroxylase (C4H) and catechins (flavan-3-ols) accumulation in tea. Funct. Integr. Genomics 9, 125–134. doi: 10.1007/s10142-008-0092-9 18679731

[B32] Sun J. Qiu C. Ding Y. Wang Y. Sun L. Fan K.. (2020). Fulvic acid ameliorates drought stress-induced damage in tea plants by regulating the ascorbate metabolism and flavonoids biosynthesis. BMC Genomics 21. doi: 10.1186/s12864-020-06815-4 PMC7301537 32552744

[B33] Thankappan N. (2023). Evaluating the effects of climate change on tea and sustainable livelihood: A study of Sri Lankan tea plantation labours’. Nisha Thankappan - IJFMR 5. doi: 10.36948/ijfmr.2023.v05i06.9652

[B34] Upadhyaya H. Panda S. K. Dutta B. K. (2008). Variation of physiological and antioxidative responses in tea cultivars subjected to elevated water stress followed by rehydration recovery. Acta Physiologiae Plantarum 30, 457–468. doi: 10.1007/s11738-008-0143-9

[B35] Upadhyaya H. Panda S. K. Dutta B. K. (2011). CaCl2 improves post-drought recovery potential in Camellia sinensis (L) O. Kuntze. Plant Cell Rep. 30, 495–503. doi: 10.1007/s00299-010-0958-x 21153899

[B36] van Eck N. J. Waltman L. (2010). Software survey: VOSviewer, a computer program for bibliometric mapping. Scientometrics 84, 523–538. doi: 10.1007/s11192-009-0146-3 20585380 PMC2883932

[B37] Wang W. Xin H. Wang M. Ma Q. Wang L. Kaleri N. A.. (2016). Transcriptomic analysis reveals the molecular mechanisms of drought-stress-induced decreases in Camellia sinensis leaf quality. Front. Plant Sci. 7. doi: 10.3389/fpls.2016.00385 PMC4811933 27066035

[B38] Wang Y.-X. Liu Z.-W. Wu Z.-J. Li H. Wang W.-L. Cui X.. (2018). Genome-wide identification and expression analysis of GRAS family transcription factors in tea plant (Camellia sinensis). Sci. Rep. 8. doi: 10.1038/s41598-018-22275-z PMC5834537 29500448

[B39] Wang S. Li T. Zheng Z. Chen H. Y. H. (2019). Soil aggregate-associated bacterial metabolic activity and community structure in different aged tea plantations. Sci. Total Environ. 654, 1023–1032. doi: 10.1016/j.scitotenv.2018.11.032 30841376

[B40] Wang Y. Yao Z. Pan Z. Wang R. Yan G. Liu C.. (2020). Tea-planted soils as global hotspots for N2O emissions from croplands. Environ. Res. Lett. 15. doi: 10.1088/1748-9326/aba5b2

[B41] Wang P. Tang J. Yuping M. Wu D. Yang J. Jin Z.. (2021). Mapping threats of spring frost damage to tea plants using satellite-based minimum temperature estimation in China. Remote. Sens. 13 p, 2713. doi: 10.3390/rs13142713

[B42] Wijeratne M. A. Anandacoomaraswamy A. Amarathunga M. K. S. L. D. Ratnasiri J. Basnayake B. R. S. B. Kalra N. (2007). Assessment of impact of climate change on productivity of tea (Camellia sinensis L.) plantations in Sri Lanka. J. Natl. Sci. Foundation Sri Lanka 35, 119–126. doi: 10.4038/jnsfsr.v35i2.3676

[B43] Yan F. Qu D. Chen X. Yang J. Zeng H. Li X. (2023). Transcriptome analysis of 5-aminolevulinic acid contributing to cold tolerance in tea leaves (Camellia sinensis L.). Forests. 14, 198. doi: 10.3390/f14020198

[B44] Yang Y.-Z. Li T. Teng R. Han M. Jing Z. (2021). Low temperature effects on carotenoids biosynthesis in the leaves of green and albino tea plant (Camellia sinensis (L.) O. Kuntze). Scientia Hortic. 285, 110164. doi: 10.1016/J.SCIENTA.2021.110164

[B45] Zhang Q. Cai M. Yu X. Wang L. Guo C. Ming R.. (2017). Transcriptome dynamics of Camellia sinensis in response to continuous salinity and drought stress. Tree Genet. Genomes 13. doi: 10.1007/s11295-017-1161-9

[B46] Zhao C. Nawaz G. Cao Q. Xu T. (2022). Melatonin is a potential target for improving horticultural crop resistance to abiotic stress. Scientia Hortic. 291, 110560. doi: 10.1016/j.scienta.2021.110560

[B47] Zheng C. Wang Y. Ding Z. Zhao L. (2016). Global transcriptional analysis reveals the complex relationship between tea quality, leaf senescence and the responses to cold-drought combined stress in Camellia sinensis. Front. Plant Sci. 7. doi: 10.3389/fpls.2016.01858 PMC5145883 28018394

[B48] Zhou L. Xu H. Mischke S. Meinhardt L. W. Zhang D. Zhu X.. (2014). Exogenous abscisic acid significantly affects proteome in tea plant (Camellia sinensis) exposed to drought stress. Horticulture Res. 1. doi: 10.1038/hortres.2014.29 PMC4816387 27076915

[B49] Zou Y. Hirono Y. Yanai Y. Hattori S. Toyoda S. Yoshida N. (2014). Isotopomer analysis of nitrous oxide accumulated in soil cultivated with tea (Camellia sinensis) in Shizuoka, central Japan. Soil Biol. Biochem. 77, 276–291. doi: 10.1016/j.soilbio.2014.06.016

