# Supplementary material for: Comprehensive review of mapping climate change impacts on tea cultivation: bibliometric and content analysis of trends, influences, adaptation strategies, and future directions
Source: Front Plant Sci. 2025 Jan 24;15:1542793. doi: 10.3389/fpls.2024.1542793 (PMC11802803; doi:10.3389/fpls.2024.1542793)
Supplement: Supplementary file 1 [file Table1.docx]

Supplementary Material

**Supplementary Table 1.** Primary climate variables impacting tea cultivation across countries and adaptation strategies for climate change mitigation

| **No.** | **Study Citation** | **Location (Country & Weather)** | **Climate Parameters** | **Climate Characteristics** | **Primary Climate Variables Impacting Tea Cultivation** | **Adaptation Strategies** | **Key Insight** |
| --- | --- | --- | --- | --- | --- | --- | --- |
| 1 | (Singh et al., 2009) | India, Subtropical | Temperature, drought stress, hormonal influences (ABA, GA_3_) | Average temperature: 22.5°C during active growth; drought stress influences catechin levels | Drought stress, temperature fluctuations, hormonal changes (ABA, GA3) | Modulation of PAL and C_4_H gene expression; wounding to enhance catechin production | PAL and C_4_H gene expressions critically impact catechin biosynthesis under stress |
| 2 | (Li et al., 2019) | China, Temperate and Subtropical climates | Cold, salt, drought stress, oxidative stress | Cold stress (4°C), high salt (300 mM NaCl), and drought stress (200 g/L PEG); antioxidant enzyme and ROS activity | Cold stress, salinity, drought, oxidative stress | Application of 100 µM melatonin; upregulation of antioxidant systems | Melatonin significantly mitigates oxidative damage and enhances photosynthesis efficiency through the regulation of ROS and antioxidant activity |
| 3 | (Wang et al., 2016) | China, Temperate and Subtropical regions | Drought stress, water scarcity, gene expression alterations | Drought-induced decreases in water content, chlorophyll levels, and increases in MDA; reduced catechin, caffeine, and theanine levels | Drought stress affecting major bioactive components in leaves | Molecular profiling for secondary metabolite pathways using RNA-Seq | RNA-Seq unveiled differential gene expressions impacting secondary metabolism pathways essential for leaf quality |
| 4 | (Ahmed et al., 2014) | China, Tropical (Yunnan Province) | Precipitation variability, seasonal droughts, monsoon onset | Annual precipitation 1,400–1,500 mm; dry spring drought; increased precipitation during monsoon | Seasonal precipitation variations affecting leaf quality and secondary metabolite concentrations | Use of traditional management practices like agroforestry and maintaining forest buffers | Seasonal precipitation inversely impacts tea leaf quality and yields; extreme weather affects farmer livelihoods |
| 5 | (Ahmed et al., 2019) | Multi-country (Global Tea Regions) | Seasonality, water stress, geography, light factors, altitude, soil and nutrient factors | Seasonal precipitation shifts, drought stress, altitude variation, solar radiation differences | Seasonality, drought stress, altitude-related temperature differences, soil nutrient availability | Agroecological diversification, breeding climate-resilient cultivars | Climate-related variability in phenolic and antioxidant compounds highlights both beneficial and detrimental effects depending on regional conditions |
| 6 | (Gai et al., 2020) | China, Subtropical (Experimental settings) | Drought stress, hormonal regulation (exogenous ABA application) | Drought-induced decreases in water and chlorophyll content, increased flavonoids, altered lipid metabolism | Drought stress, water scarcity, biochemical imbalances | Application of exogenous ABA to regulate lipid and flavonoid metabolism | Exogenous ABA effectively alleviates drought damage by maintaining metabolic balance, enhancing flavonoid production, and regulating gene expression for drought response |
| 7 | (Jayasinghe and Kumar, 2019) | Sri Lanka, Tropical | Temperature, precipitation, seasonality | Mean annual temperature: 27°C (coastal) to 16°C (highlands); annual precipitation: 900–5000 mm | Precipitation seasonality, temperature seasonality, mean annual temperature, and annual precipitation | Shifting cultivation zones to higher altitudes; targeted land resource planning using species distribution models (SDMs) | Climate change significantly reduces optimal and medium climate suitability areas for tea; high-elevation areas fare better than lowlands |
| 8 | (Zhou et al., 2014) | China, Subtropical (Greenhouse) | Drought stress, hormonal influence (ABA application) | Controlled temperature (15–20°C), humidity (60–70%), drought induced using 10% PEG solution | Drought-induced oxidative stress, proteomic changes, alterations in photosynthesis and metabolism | Exogenous ABA application to enhance protein transport and antioxidant activity | Exogenous ABA pre-treatment enhances drought resilience by upregulating proteins involved in photosynthesis, ROS control, and carbon metabolism |
| 9 | (Cheruiyot et al., 2007) | Kenya, Subtropical (Rain-out shelter) | Soil water content (SWC), drought stress | Declining SWC levels (38%, 30%, 22%, 14%) impact polyphenols and shoot growth; drought-tolerant clones showed stable polyphenol levels | Soil water content, drought-induced polyphenol variability | Selection of drought-tolerant clones with high polyphenol stability | Polyphenols correlated with drought tolerance and shoot growth; clones with stable polyphenol levels are drought-tolerant |
| 10 | (Hernández et al., 2006) | Spain, Temperate | Water deficit, vapor pressure deficit (VPD), antioxidant responses | Max temperature: 31°C; min: 16.4°C; severe water deficit led to reduced relative leaf water content (50%) and increased oxidative stress markers | Severe water deficit, high VPD, increased flavonoid oxidation | Management of flavonoid oxidation and proanthocyanidin biosynthesis | Water stress enhances flavonoid oxidation and proanthocyanidin biosynthesis, reducing oxidative damage and lipid peroxidation |
| 11 | (Wang et al., 2019) | China, Subtropical (Western Sichuan) | Soil aggregate fractions, bacterial activity, organic carbon levels | Annual temperature: 15.4°C; annual rainfall: 1500 mm; soil pH declines over plantation age | Soil organic matter (SOM), soil pH, bacterial metabolic activity | Maintaining soil microbial diversity via sustainable management practices | Soil aggregate-associated bacterial metabolic activity and community diversity peaked in 23-year-old plantations; SOM and soil pH were key drivers of community structure |
| 12 | (Upadhyaya et al., 2008) | India, Subtropical (North-East) | Drought stress, rehydration recovery | Temperature: 25.1–32.3°C max/min; RH: 38–96%; drought duration: 20 days | Drought-induced oxidative stress, reactive oxygen species (ROS) activity | Selection of drought-tolerant clones based on antioxidative efficiency | Differential antioxidative enzyme responses (SOD, CAT, POX) in tea clones indicate drought resilience potential; TV-1 and TV-30 exhibit higher recovery rates post-rehydration |
| 13 | (Liu et al., 2016) | China, Subtropical | Drought stress, rehydration dynamics | Drought reduced soil moisture content to ~12.5%; recovery stage after 8 days | Soil water content, transcriptional responses, stress-related hormones | Utilization of transcriptomics for breeding drought-resistant cultivars | Differential expression of 5,955 genes during drought and recovery phases highlights complex regulatory networks |
| 14 | (Li et al., 2017) | China, Subtropical (Controlled environment) | Elevated CO_2_ concentration (800 µmol mol−1) | Enhanced photosynthesis, biomass accumulation, decreased nitrogen levels, elevated sugar, starch, and polyphenol content | Elevated CO_2_ affecting carbon to nitrogen balance, photosynthetic acclimation | Exploiting elevated CO_2_ conditions to improve tea quality | Elevated CO_2_ increased catechins and theanine while reducing caffeine content; biosynthetic genes of catechins and theanine upregulated under CO_2_ enrichment |
| 15 | (Guo et al., 2017) | China, Subtropical | Drought stress, miRNA expression regulation | Reduced leaf water content (18.86% decrease under severe drought), increased malondialdehyde concentration, decreased chlorophyll | Drought stress-induced miRNA and transcriptional changes | Utilizing miRNA profiling for identifying drought-resistant cultivars | Differentially expressed miRNAs are crucial in regulating transcription and metabolic pathways like sulfur metabolism, which help mitigate drought stress |
| 16 | (Wang et al., 2018) | China, Subtropical (Controlled environment) | Abiotic stresses (salt, drought, cold, heat), gibberellin treatment | Salt: 200 mM NaCl; drought: 20% PEG; cold: 4°C; heat: 38°C; variable gene expression across treatments | Abiotic stresses influencing GRAS gene activity and signalling pathways | Targeting GRAS genes for improving abiotic stress tolerance | GRAS transcription factors exhibit tissue-specific and stress-induced differential expression patterns, highlighting potential as molecular targets |
| 17 | (Zhang et al., 2017) | China (Fujian Province, controlled stress simulation) | Salinity, drought | Simulated stress with polyethylene glycol and NaCl solutions | Drought and salinity were identified as key stressors influencing metabolic and transcriptomic responses | Enhancing proline, GABA, and polyphenol pathways for resilience | Uncovered overlapping and unique transcriptomic changes under salinity and drought stress |
| 18 | (Han et al., 2017) | China, Subtropical (Lushan Mountain) | Altitudinal gradients, temperature variations, rainfall | Altitude range: 212–1020 m; temperatures decreased with altitude; rainfall increased with altitude | Altitudinal gradient effects on temperature, rainfall, and chemical composition | Developing cultivation strategies for high-altitude regions to balance yield and quality | Polyphenols decreased, while amino acids (including theanine) increased with altitude; improved tea quality but lower yield in higher altitudes due to reduced polyphenol-to-amino acid ratio |
| 19 | (Zheng et al., 2016) | China, Subtropical (Arid and Semi-Arid Areas) | Cold and drought stress (combined), RNA-Seq transcriptional profiling | Cold acclimation at 15/10°C and chilling at 4/2°C combined with drought stress (15 days of no watering) | Combined stress responses, transcriptomic shifts, gene expression alterations | Identifying and modulating genes with roles in antioxidant capacity and stress adaptation | Unique transcriptional patterns during combined stress highlighted adaptive genes; flavonoids and antioxidant pathways enhance tea quality and stress tolerance |
| 20 | (Singh et al., 2008) | India, Subtropical (Controlled Environment) | Drought stress, ABA, GA_3_ treatment, wounding | Temp: 25 ± 1°C; RH: 70–80%; Wounding enhanced catechins; drought, ABA, and GA3 reduced catechins | Environmental stresses impacting flavonoid biosynthesis pathways | Modulation of flavonoid biosynthesis through external stimuli | F_3_H expression positively correlates with catechin concentration; the substrate feedback mechanism regulates catechin synthesis under stress |
| 21 | (Sun et al., 2020) | China, Subtropical (Controlled environment) | Drought stress, fulvic acid (FA) treatment | Severe drought: soil water capacity reduced to 15–25%; mild drought: 65–75% | Drought-induced oxidative stress, ROS accumulation, decreased leaf water content (LWC) | Application of fulvic acid to enhance antioxidant pathways | Fulvic acid significantly improves drought resilience by enhancing ascorbate metabolism, glutathione metabolism, and flavonoid biosynthesis |
| 22 | (Wijeratne et al., 2007) | Sri Lanka, Tropical Monsoon | Temperature, rainfall, CO_2_ concentration | Temp: 15.5–32°C depending on altitude; Rainfall: 1000–4000 mm; CO_2_ increase projected to 600 ppm | Increased temperature and reduced rainfall lead to yield declines; CO_2_ enrichment increases yield by 33-37% | Adaptation strategies include shade trees, soil conservation, drought-resistant cultivars | CO_2_ enrichment benefits productivity, but high temperatures and dry conditions limit this effect at lower elevations |
| 23 | (Gu et al., 2020) | China, Subtropical (Controlled environment) | Drought stress, lignin, flavonoids, fatty acids biosynthesis | Temp: 25°C light, 20°C dark; 75% RH; Drought imposed by withholding water for 96 h | Drought-induced biochemical changes in flavonoid, lignin, and fatty acid metabolism | Modulation of flavonoid and lignin biosynthesis to enhance stress tolerance | Drought stress enhances lignin and flavonoid content by downregulating their biosynthetic enzymes, improving drought resilience |
| 24 | (Zou et al., 2014) | China, Multi-environment (field and greenhouse) | Heat, drought, oxidative stress; tea polyphenols as protective agents | Heat-induced increase in ROS; drought lowers photosynthesis efficiency; protective effects of exogenous polyphenols | Drought, heat, ROS accumulation | Application of tea polyphenols as natural stress protectants | Exogenous polyphenols significantly reduced ROS accumulation, improved enzymatic antioxidants, and enhanced plant growth under stress |
| 25 | (Upadhyaya et al., 2011) | India, Subtropical | Drought stress, rehydration recovery, foliar calcium chloride (CaCl_2_) | Drought duration: 20 days; rehydration improved with 50–100 µM CaCl_2_ foliar spray | Drought-induced oxidative stress, reduced RWC, lipid peroxidation | Foliar spray of CaCl_2_ to enhance enzymatic and non-enzymatic antioxidant activities | CaCl_2_ significantly enhances antioxidative responses (SOD, CAT, POX, GR) and phenolic content, reducing ROS and improving drought recovery |

**Supplementary Table 2.** Emerging trends, knowledge gaps, and future research directions in tea cultivation under climate stress

| **No.** | **Study Citation** | **Emerging Trends in Tea Cultivation** | **Knowledge Gaps in Tea Cultivation** | **Future Research Directions** |
| --- | --- | --- | --- | --- |
| 1 | (Singh et al., 2009) | Gene-focused studies for catechin biosynthesis regulation; linking phenylpropanoid and flavonoid pathways | Limited understanding of gene-environment interaction for catechin synthesis under stress | Identifying molecular targets for improving drought resistance; exploring broader environmental stress markers |
| 2 | (Li et al., 2019) | Use of melatonin in improving abiotic stress tolerance | Insufficient understanding of melatonin's long-term effects on plant yield and stress response | Detailed exploration of molecular mechanisms of melatonin in stress resilience; long-term field studies on tea yield and quality |
| 3 | (Wang et al., 2016) | Transcriptomic data identified key genes in flavonoid, caffeine, and theanine biosynthetic pathways under stress | Insufficient field-based validation of transcriptomic insights; integration of metabolomics and phenotypic data needed | Combining transcriptomics, metabolomics, and phenotypic studies to optimize tea plant resilience under varying climatic stressors |
| 4 | (Ahmed et al., 2014) | Agroecological knowledge contributing to climate adaptation | Lack of multi-seasonal data on precipitation effects; limited insight into long-term impacts of farmer adaptation practices | Expanding research to evaluate the long-term impacts of adaptive practices, scaling findings across diverse geographies and climatic zones |
| 5 | (Ahmed et al., 2019) | Use of systematic reviews to identify trends in tea quality variability | Contradictory findings on soil fertility and herbivory; insufficient long-term carbon dioxide studies | Developing standardized protocols for quality assessment; modeling system-wide impacts of environmental shifts on tea quality |
| 6 | (Gai et al., 2020) | Transcriptomics and metabolomics approaches to enhance drought resistance in tea | Limited understanding of ABA's broader regulatory roles in field conditions; insufficient linkage of findings with long-term yield impacts | Expanding metabolomics studies to diverse stress scenarios; integrating ABA studies with real-field climatic conditions |
| 7 | (Jayasinghe and Kumar, 2019) | Use of MaxEnt models for species distribution; emphasis on dynamic suitability mapping under climate change scenarios | Lack of long-term field studies validating MaxEnt outputs; insufficient adaptation strategies for specific agroecological zones | Incorporating socioeconomic factors into climate suitability models; expanding SDM studies across tropical regions to predict tea yield impacts |
| 8 | (Zhou et al., 2014) | Proteomic analysis of drought and ABA-responsive proteins in tea plants | Insufficient field trials for ABA efficacy under natural drought conditions; limited data on long-term yield impacts | Translating proteomic findings to field conditions; developing cost-effective ABA applications for large-scale agriculture |
| 9 | (Cheruiyot et al., 2007) | Using polyphenols as biochemical markers for drought tolerance in tea plants | Lack of field validation for polyphenol-based drought tolerance markers; insufficient study on specific flavonoid derivatives | Investigating specific flavonoids (e.g., catechins) as advanced markers for drought tolerance; expanding studies to diverse environmental conditions |
| 10 | (Hernández et al., 2006) | Antioxidant-mediated protection mechanisms under stress | Lack of understanding of the biochemical pathways for flavonoid oxidation; insufficient field trials for stress adaptation | Investigating proanthocyanidin biosynthesis mechanisms under diverse stress conditions; exploring long-term stress resilience markers |
| 11 | (Wang et al., 2019) | Emphasis on understanding long-term microbial dynamics in tea ecosystems | Lack of data on the interaction between microbial communities and soil aggregate properties across different climates | Expansion of microbial metagenomics and proteomics to study interactions between microbial activity and soil aggregates globally |
| 12 | (Upadhyaya et al., 2008) | Antioxidative and physiological markers as tools for evaluating drought resilience | Limited understanding of molecular mechanisms linking oxidative damage and recovery; insufficient field validation of antioxidant roles | Expanding recovery studies to correlate physiological markers with yield; integrating drought response findings into breeding programs |
| 13 | (Liu et al., 2016) | RNA-Seq revealed extensive transcriptional shifts during stress and recovery | Limited insight into field-applicable responses; insufficient connection to long-term yield and tea quality | Expansion of transcriptomic studies to natural field settings; breeding programs integrating molecular insights |
| 14 | (Li et al., 2017) | Molecular insights into gene regulation of secondary metabolites under elevated CO_2_ | Limited understanding of the long-term impacts of CO_2_ on field-grown plants; insufficient linkage between lab and field outcomes | Long-term studies on elevated CO_2_ impacts on yield and stress resilience; optimizing tea quality through CO_2_ modulation |
| 15 | (Guo et al., 2017) | Phase-specific miRNAs revealed; miRNA-mRNA regulatory networks for drought adaptation explored | Limited understanding of the phase-specific miRNA roles; insufficient real-world trials of miRNA-guided drought adaptation strategies | Expanding studies on miRNA-target interactions; integrating miRNA profiling into breeding programs for drought resistance |
| 16 | (Wang et al., 2018) | GRAS family transcription factors play diverse roles in development and stress responses | Limited field-based validation of GRAS gene functions under stress; insufficient linkage to yield improvement | Expanding the functional characterization of GRAS genes under field stress; integrating findings into molecular breeding programs |
| 17 | (Zhang et al., 2017) | Molecular identification of DEGs under salt and drought stress | Limited field-based validation of transcriptomic findings and long-term adaptation mechanisms | Field-level transcriptomic validation and integration with socioeconomic and climatic data |
| 18 | (Han et al., 2017) | Understanding interactions between climate and chemical profiles in tea at varying altitudes | Insufficient data on long-term climate impacts on chemical composition; limited scalability of high-altitude cultivation strategies | Expanding research on altitudinal impacts across different geographic regions; integrating climate data to predict quality changes under global warming |
| 19 | (Zheng et al., 2016) | RNA-Seq revealed co-regulation of stress-specific genes under combined cold and drought stress | Limited understanding of interactions between cold and drought responses in field-grown plants; insufficient application of transcriptomic findings in breeding | Expanding research on combined stress impacts in natural environments; identifying genes with potential for genetic engineering of dual-stress-resistant cultivars |
| 20 | (Singh et al., 2008) | Using flavanone 3-hydroxylase (F_3_H) as a marker for catechin synthesis | Limited understanding of feedback inhibition and real-field responses; insufficient field studies validating catechin-F3H relationships | Expanding studies on the role of F3H in stress environments; integrating findings into molecular breeding for enhanced tea quality |
| 21 | (Sun et al., 2020) | Utilization of transcriptomics and metabolomics to understand stress responses | Insufficient field trials to validate laboratory findings; limited understanding of FA impacts on long-term yield | Expanding metabolomics and transcriptomics analysis to field conditions; scaling FA applications in real-world tea cultivation systems |
| 22 | (Wijeratne et al., 2007) | Crop modeling predicts yield increases at high elevations, reductions at low elevations | Lack of field data on combined climatic impacts; limited application of crop models in real farming systems | Expanding crop modeling studies across diverse tea-growing regions; integrating economic feasibility analyses for adaptation strategies |
| 23 | (Gu et al., 2020) | Proteomic analysis reveals downregulation of key biosynthetic enzymes for flavonoids and lignin under drought | Insufficient understanding of the molecular regulation of flavonoid and lignin biosynthesis under drought; limited validation in field conditions | Expanding proteomic studies to field scenarios; exploring combinatory stress effects and their impact on secondary metabolite pathways |
| 24 | (Zou et al., 2014) | Exploring multifunctional roles of tea polyphenols for improving tolerance and crop quality | Insufficient understanding of the long-term effects of polyphenols under varying environmental stresses; scalability issues for polyphenol application | Investigating broader environmental impacts; field-based validation for exogenous polyphenols in diverse cropping systems |
| 25 | (Upadhyaya et al., 2011) | Exploring calcium's role in abiotic stress recovery | Insufficient understanding of calcium-induced gene regulation and field-level applications of CaCl_2_ | Exploring calcium signaling in antioxidant pathways; field validation for large-scale CaCl_2_ applications in tea plantations |

**References**

Ahmed, S., Griffin, T. S., Kraner, D., Schaffner, M. K., Sharma, D., Hazel, M., et al. (2019). Environmental Factors Variably Impact Tea Secondary Metabolites in the Context of Climate Change. *Front. Plant Sci.* 10. doi: 10.3389/fpls.2019.00939

Ahmed, S., Stepp, J. R., Orians, C., Griffin, T., Matyas, C., Robbat, A., et al. (2014). Effects of extreme climate events on tea (Camellia sinensis) functional quality validate indigenous farmer knowledge and sensory preferences in Tropical China. *PLoS One* 9. doi: 10.1371/journal.pone.0109126

Cheruiyot, E. K., Mumera, L. M., Ng’etich, W. K., Hassanali, A., and Wachira, F. (2007). Polyphenols as potential indicators for drought tolerance in tea (Camellia sinensis L.). *Biosci. Biotechnol. Biochem.* 71, 2190–2197. doi: 10.1271/bbb.70156

Gai, Z., Wang, Y., Ding, Y., Qian, W., Qiu, C., Xie, H., et al. (2020). Exogenous abscisic acid induces the lipid and flavonoid metabolism of tea plants under drought stress. *Sci. Rep.* 10. doi: 10.1038/s41598-020-69080-1

Gu, H., Wang, Y., Xie, H., Qiu, C., Zhang, S., Xiao, J., et al. (2020). Drought stress triggers proteomic changes involving lignin, flavonoids and fatty acids in tea plants. *Sci. Rep.* 10. doi: 10.1038/s41598-020-72596-1

Guo, Y., Zhao, S., Zhu, C., Chang, X., Yue, C., Wang, Z., et al. (2017). Identification of drought-responsive miRNAs and physiological characterization of tea plant (Camellia sinensis L.) under drought stress. *BMC Plant Biol.* 17. doi: 10.1186/s12870-017-1172-6

Han, W.-Y., Huang, J.-G., Li, X., Li, Z.-X., Ahammed, G. J., Yan, P., et al. (2017). Altitudinal effects on the quality of green tea in east China: a climate change perspective. *Eur. Food Res. Technol.* 243, 323–330. doi: 10.1007/s00217-016-2746-5

Hernández, I., Alegre, L., and Munné-Bosch, S. (2006). Enhanced oxidation of flavan-3-ols and proanthocyanidin accumulation in water-stressed tea plants. *Phytochemistry* 67, 1120–1126. doi: 10.1016/j.phytochem.2006.04.002

Jayasinghe, S. L., and Kumar, L. (2019). Modeling the climate suitability of tea [Camellia sinensis(L.) O. Kuntze] in Sri Lanka in response to current and future climate change scenarios. *Agric. For. Meteorol.* 272–273, 102–117. doi: 10.1016/j.agrformet.2019.03.025

Li, J., Yang, Y., Sun, K., Chen, Y., Chen, X., and Li, X. (2019). Exogenous melatonin enhances cold, salt and drought stress tolerance by improving antioxidant defense in tea plant (Camellia sinensis (L.) O. Kuntze). *Molecules* 24. doi: 10.3390/molecules24091826

Li, X., Zhang, L., Ahammed, G. J., Li, Z.-X., Wei, J.-P., Shen, C., et al. (2017). Stimulation in primary and secondary metabolism by elevated carbon dioxide alters green tea quality in Camellia sinensis L. *Sci. Rep.* 7. doi: 10.1038/s41598-017-08465-1

Liu, S.-C., Jin, J.-Q., Ma, J.-Q., Yao, M.-Z., Ma, C.-L., Li, C.-F., et al. (2016). Transcriptomic analysis of tea plant responding to drought stress and recovery. *PLoS One* 11. doi: 10.1371/journal.pone.0147306

Singh, K., Kumar, S., Rani, A., Gulati, A., and Ahuja, P. S. (2009). Phenylalanine ammonia-lyase (PAL) and cinnamate 4-hydroxylase (C4H) and catechins (flavan-3-ols) accumulation in tea. *Funct. Integr. Genomics* 9, 125–134. doi: 10.1007/s10142-008-0092-9

Singh, K., Rani, A., Kumar, S., Sood, P., Mahajan, M., Yadav, S. K., et al. (2008). An early gene of the flavonoid pathway, flavanone 3-hydroxylase, exhibits a positive relationship with the concentration of catechins in tea (Camellia sinensis). *Tree Physiol.* 28, 1349–1356. doi: 10.1093/treephys/28.9.1349

Sun, J., Qiu, C., Ding, Y., Wang, Y., Sun, L., Fan, K., et al. (2020). Fulvic acid ameliorates drought stress-induced damage in tea plants by regulating the ascorbate metabolism and flavonoids biosynthesis. *BMC Genomics* 21. doi: 10.1186/s12864-020-06815-4

Upadhyaya, H., Panda, S. K., and Dutta, B. K. (2008). Variation of physiological and antioxidative responses in tea cultivars subjected to elevated water stress followed by rehydration recovery. *Acta Physiol. Plant.* 30, 457–468. doi: 10.1007/s11738-008-0143-9

Upadhyaya, H., Panda, S. K., and Dutta, B. K. (2011). CaCl2 improves post-drought recovery potential in Camellia sinensis (L) O. Kuntze. *Plant Cell Rep.* 30, 495–503. doi: 10.1007/s00299-010-0958-x

Wang, S., Li, T., Zheng, Z., and Chen, H. Y. H. (2019). Soil aggregate-associated bacterial metabolic activity and community structure in different aged tea plantations. *Sci. Total Environ.* 654, 1023–1032. doi: 10.1016/j.scitotenv.2018.11.032

Wang, W., Xin, H., Wang, M., Ma, Q., Wang, L., Kaleri, N. A., et al. (2016). Transcriptomic analysis reveals the molecular mechanisms of drought-stress-induced decreases in Camellia sinensis leaf quality. *Front. Plant Sci.* 7. doi: 10.3389/fpls.2016.00385

Wang, Y.-X., Liu, Z.-W., Wu, Z.-J., Li, H., Wang, W.-L., Cui, X., et al. (2018). Genome-wide identification and expression analysis of GRAS family transcription factors in tea plant (Camellia sinensis). *Sci. Rep.* 8. doi: 10.1038/s41598-018-22275-z

Wijeratne, M. A., Anandacoomaraswamy, A., Amarathunga, M. K. S. L. D., Ratnasiri, J., Basnayake, B. R. S. B., and Kalra, N. (2007). Assessment of impact of climate change on productivity of tea (Camellia sinensis L.) plantations in Sri Lanka. *J. Natl. Sci. Found. Sri Lanka* 35, 119–126. doi: 10.4038/jnsfsr.v35i2.3676

Zhang, Q., Cai, M., Yu, X., Wang, L., Guo, C., Ming, R., et al. (2017). Transcriptome dynamics of Camellia sinensis in response to continuous salinity and drought stress. *Tree Genet. Genomes* 13. doi: 10.1007/s11295-017-1161-9

Zheng, C., Wang, Y., Ding, Z., and Zhao, L. (2016). Global transcriptional analysis reveals the complex relationship between tea quality, leaf senescence and the responses to cold-drought combined stress in Camellia sinensis. *Front. Plant Sci.* 7. doi: 10.3389/fpls.2016.01858

Zhou, L., Xu, H., Mischke, S., Meinhardt, L. W., Zhang, D., Zhu, X., et al. (2014). Exogenous abscisic acid significantly affects proteome in tea plant (Camellia sinensis) exposed to drought stress. *Hortic. Res.* 1. doi: 10.1038/hortres.2014.29

Zou, Y., Hirono, Y., Yanai, Y., Hattori, S., Toyoda, S., and Yoshida, N. (2014). Isotopomer analysis of nitrous oxide accumulated in soil cultivated with tea (Camellia sinensis) in Shizuoka, central Japan. *Soil Biol. Biochem.* 77, 276–291. doi: 10.1016/j.soilbio.2014.06.016
